# Co-transmission of acetylcholine and GABA regulates hippocampal states

**DOI:** 10.1038/s41467-018-05136-1

**Published:** 2018-07-20

**Authors:** Virág T. Takács, Csaba Cserép, Dániel Schlingloff, Balázs Pósfai, András Szőnyi, Katalin E. Sos, Zsuzsanna Környei, Ádám Dénes, Attila I. Gulyás, Tamás F. Freund, Gábor Nyiri

**Affiliations:** 1 Laboratory of Cerebral Cortex Research Institute of Experimental Medicine, Hungarian Academy of Sciences, Szigony u 43, Budapest, 1083 Hungary; 2 János Szentágothai Doctoral School of Neurosciences, Semmelweis University, Budapest, 1085 Hungary; 3 Present Address: Momentum Laboratory of Neuroimmunology, Institute of Experimental Medicine, Hungarian Academy of Sciences, Szigony u 43, Budapest, 1083 Hungary; 4 Momentum Laboratory of Neuroimmunology, Institute of Experimental Medicine, Hungarian Academy of Sciences, Szigony u 43, Budapest, 1083 Hungary

## Abstract

The basal forebrain cholinergic system is widely assumed to control cortical functions via non-synaptic transmission of a single neurotransmitter. Yet, we find that mouse hippocampal cholinergic terminals invariably establish GABAergic synapses, and their cholinergic vesicles dock at those synapses only. We demonstrate that these synapses do not co-release but co-transmit GABA and acetylcholine via different vesicles, whose release is triggered by distinct calcium channels. This co-transmission evokes composite postsynaptic potentials, which are mutually cross-regulated by presynaptic autoreceptors. Although postsynaptic cholinergic receptor distribution cannot be investigated, their response latencies suggest a focal, intra- and/or peri-synaptic localisation, while GABA_A_ receptors are detected intra-synaptically. The GABAergic component alone effectively suppresses hippocampal sharp wave-ripples and epileptiform activity. Therefore, the differentially regulated GABAergic and cholinergic co-transmission suggests a hitherto unrecognised level of control over cortical states. This novel model of hippocampal cholinergic neurotransmission may lead to alternative pharmacotherapies after cholinergic deinnervation seen in neurodegenerative disorders.

## Abstract (web summary)

Acetylcholine (ACh) release in the central nervous system is thought to be unitary and mediated non-synaptically in volume transmission. Here, Takács and colleagues show cholinergic terminals juxtapose GABAergic synapses anatomically and functionally, and GABA and ACh molecules are co-transmitted.

## Introduction

The cholinergic system arising from the basal forebrain^1,2^ has a fundamental role in controlling cortical functions including attention^3^, learning and memory^4^, plasticity^5^, sleep–wake alternation^6^, and is implicated in neurodegenerative diseases^7^.

Contemporary models of the basal forebrain cholinergic system and efforts to develop pro-cholinergic treatments have been based largely on the assumption that cholinergic cells release only a single transmitter and it is released non-synaptically^8–13^. The seemingly rare synapses on cholinergic fibres (see Supplementary Discussion) supported the concept of non-synaptic transmission. However, highly precise cholinergic transmission during reward and punishment^14^, recordings of phasic release^10,15,16^, and the dependence of hippocampal synaptic plasticity on the millisecond-scale timing of the cholinergic input^17^ challenge this textbook model of non-synaptic transmission by cholinergic fibres.

Therefore, we hypothesised that all cholinergic terminals establish synapses. After immunolabeling, we analysed the real incidence of synapses, localised vesicle pools using STORM super-resolution imaging and we also localised membrane-docked neurotransmitter vesicles using electron tomography. Because previous data suggested the co-localisation of acetylcholine and GABA in retina and other brain areas^18–23^, we also hypothesised that hippocampal cholinergic fibres may be GABAergic as well. Using immunolabelling and optogenetics combined with in vitro electrophysiology, we investigated the possible presence and subcellular regulation of hippocampal co-transmission of acetylcholine and GABA, and the role of its GABAergic component in controlling hippocampal network activity.

Challenging a decades-old model, we show that all hippocampal cholinergic terminals establish GABAergic synapses, where cholinergic vesicles are released as well, and these synapses evoke composite (hyperpolarising and depolarising) postsynaptic potentials. Our data suggest synaptic release and action of GABA and synaptic release and a focal, synaptic and/or peri-synaptic action of acetylcholine. GABA and acetylcholine transmissions are modulated by distinct calcium channels and were mutually regulated by presynaptic autoreceptors. We demonstrate here that synaptic release of GABA from cholinergic terminals alone can suppress hippocampal sharp wave-ripples effectively and it can attenuate hippocampal epileptiform activity as well.

Our data urge the re-interpretation of previous studies about the basal forebrain cholinergic system and offer a new explanation for the emergence of hippocampal epileptiform activity associated with Alzheimer’s disease-related loss of cholinergic innervation.

## Results

### All hippocampal cholinergic axon terminals form synapses

**Fig. 1:**
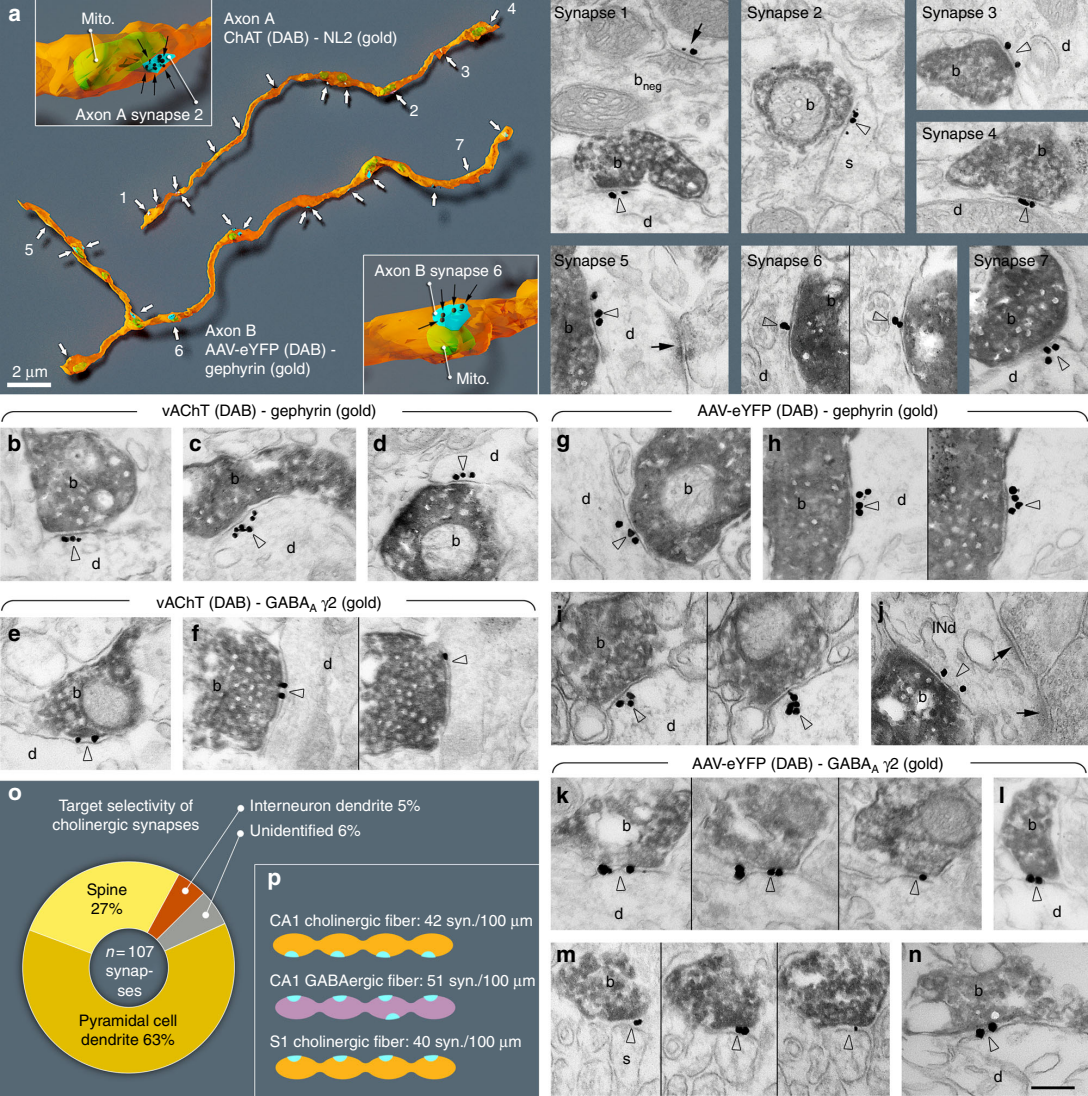
All cholinergic terminals establish synapses, express GABAergic markers and innervate pyramidal cells or interneurons. **a** Three-dimensional EM reconstructions show that hippocampal cholinergic fibres form synapses (arrows) frequently. Axon A is labelled for ChAT in a WT mouse. Axon B is an AAV-eYFP virus-labelled septo-hippocampal fibre in a ChAT-Cre mouse. Insets show two typical terminals with synapses (blue). The plasma membrane was made partially transparent to reveal mitochondria (mito, green). Gold labellings of NL2 (axon A, synapse 1–4) or gephyrin (axon B, synapse 5–7) were used to recognise synapses (black dots and arrows in the insets). EM images show terminal boutons (b) of the reconstructed axonal segments establishing synapses 1–7 (arrowheads, indicated by the same numbers on the left) on dendrites (d) and a spine (s). Next to synapse 1, a ChAT-negative, putative GABAergic terminal bouton (b_neg_) forming a NL2-positive synapse (arrow) is also shown. **b**–**n** EM images reveal the presence of gephyrin (arrowheads, gold; **b**–**d**; **g**–**j**) and GABA_A_ γ2 receptor subunits (arrowheads, gold; **e**–**f**; **k**–**n**) postsynaptically in synapses established by vAChT-positive terminals in WT mice (**b**–**f**; DAB, b) or by AAV-eYFP-labelled septo-hippocampal terminals in ChAT-Cre mice (**g**–**n**; DAB, b). Images of consecutive sections are separated by thin black lines. Terminals innervate dendrites (d) or spines (s). In **j**, the postsynaptic target is an interneuron dendrite (INd) that receives type-I synapses as well (arrows). Synapses are from str. ori. (**a**, **b**–**e**, **g**–**j**, **l**, **m**), str. rad. (**k**, **n**) and str. l-m (**f**). Scale bar is 200 nm for all EM images. **o** Postsynaptic target selectivity of reconstructed cholinergic axonal segments from str. oriens and radiatum. Spine: 27.1%, pyramidal cell dendrite: 62.6%, interneuron dendrite: 4.7%, unidentifiable: 5.6%. **p** Comparison of the number of synapses per 100 µm cholinergic axonal segments in CA1 and S1 cortex and GABAergic fibres in CA1

Previous studies concluded that few cholinergic terminals form synapses (see Discussion and Supplementary Discussion). We hypothesised that all cholinergic terminals form synapses. By identifying synapses with neuroligin 2 (NL2) labelling (Fig. 1, Supplementary Figure 1^24^,) on cholinergic fibres, we could test the incidence of synapses with three-dimensional serial electron microscopic reconstructions in the hippocampus. We reconstructed randomly selected, long axonal segments [6–33 µm, average: 21 µm, *n* = 17, labelled either with anti-choline acetyltransferase (ChAT) antibody in wild-type (WT) mice or with eYFP-adeno-associated viruses (AAV) injected in ChAT-Cre mice, Fig. 1, Supplementary Figure 1, Supplementary Table 4, for controls see Supplementary Note 7 and Supplementary Figure 2] and identified their synapses with NL2 or gephyrin immunogold labelling. All of them established synapses abundantly (Fig. 1, Supplementary Figure 1, Supplementary Note 1). The average density of synapses was 42 synapses/100 µm. Some of these contact sites would not have been considered synapses earlier, because of their weak membrane thickening and narrower intercellular synaptic gap (e.g. Figure 1a, synapse 2–3);^24^ however, NL2 and gephyrin labelling clearly identified their active zones. For comparison, we have also reconstructed GABAergic axonal segments (labelled for cannabinoid receptor type-1, CB_1_; *n* = 2, 18 and 29 µm, Supplementary Figure 1, Supplementary Table 4), which are known to establish synapses abundantly. Having verified that all hippocampal cholinergic terminals originate from basal forebrain cholinergic cells (for controls see Supplementary Note 7 and Supplementary Figure 2), we found that practically all hippocampal cholinergic terminals examined established one or more NL2-positive synapses (Fig. 1, Supplementary Figure 1; Supplementary Table 4, Supplementary Note 1). As a consequence, the linear density of synapses along cholinergic axons was similar to that along GABAergic axons (Supplementary Figure 1, Supplementary Table 4, number of synapses per 100 µm cholinergic axons: 42 in CA1, 40 in S1, number of synapses per 100 µm CB_1_-positive axons: 51).

Using electron microscopy in hippocampal CA1, we found that NL2 and gephyrin positive cholinergic synapses (*n* = 107, collected from four mice) predominantly innervated pyramidal dendritic shafts (63%) and spine-necks (27%), and they also innervated interneuron dendrites (5%), while some (6%) postsynaptic targets could not be classified (Fig. 1o, Supplementary Table 4).

All innervated spines received another, putatively glutamatergic asymmetric, type-I input from an unlabelled terminal, suggesting that, contrary to previous suggestion^25^, cholinergic synapses alone do not induce spine formation. These data suggest that about 90% of these synapses target pyramidal cells in CA1, whereas they also innervate interneurons (at least 5%), which ratio is close to the neuronal ratios in CA1.

We tested whether these synapses are GABAergic as well. First, we localised the elements of postsynaptic GABAergic signalling machinery in these contacts. We localised gephyrin first, because it is known to interact with both GABA_A_ receptors and NL2. We found, that at least 81% of synapses on hippocampal cholinergic fibres contained gephyrin postsynaptically, on dendrites and spine-necks (Fig. 1, Supplementary Note 2, Supplementary Table 3). In addition, we found that at least 80% of these synapses showed GABA_A_ receptor gamma2 subunit labelling that was also readily detected on both dendrites and spine-necks (Fig. 1, Supplementary Figure 1, Supplementary Table 3, and Supplementary Note 2).

**Fig. 2:**
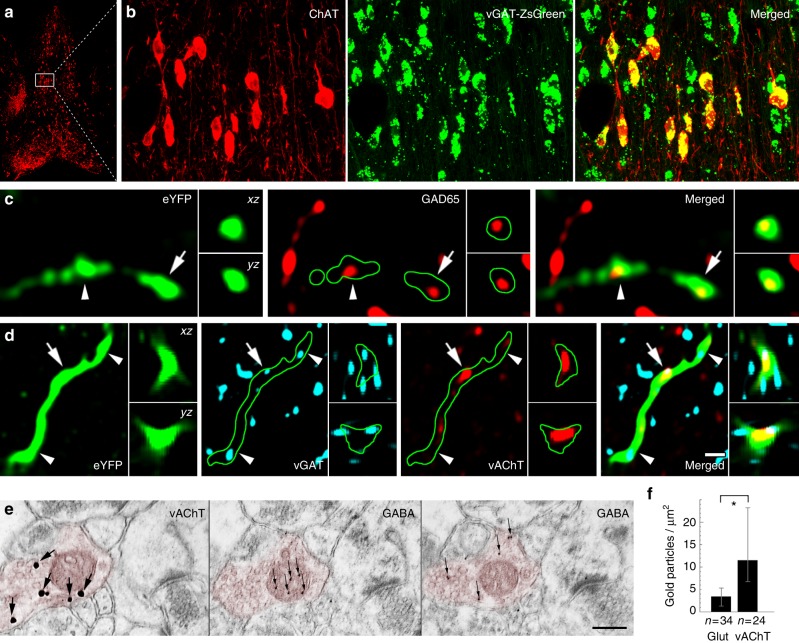
Cholinergic cells express the molecular machinery required for GABA release. **a**, **b** The cholinergic neurons of the MS are GABAergic. White box in **a** contains area enlarged in **b**. Images show neurons stained for ChAT in red, while the green labelling marks the vGAT-expressing neurons in vGAT-ZsGreen reporter mouse. **c**, **d** AAV-eYFP virus-traced septo-hippocampal fibres express GAD65 (**c**). AAV-eYFP virus-traced septo-hippocampal fibres express vGAT and vAChT (**d**). Insets show *xz* and *yz* projections of the terminal labelled with an arrow. Arrowhead points to another terminal. Green line marks the fibre outline. (Scale bar on **d** is 210, 14, 2 and 1 μm for **a**, **b**, **c** and **d**, respectively.) **e**, **f** Hippocampal cholinergic terminals contain GABA. Three consecutive EM sections of a vAChT-positive terminal (**e**, red pseudocolor) are shown. vAChT was visualised by pre-embedding immunogold method (the first panel of **e**, silver-intensified gold particles, large arrows), whereas on the next ultrathin sections (the second and third panels of **e**) postembedding GABA immunostaining was performed (smaller gold particles, thin arrows, some GABA molecules penetrate into mitochondria during fixation). vAChT signal is absent in postembedding images, because of the etching procedure. Scale bar is 200 nm for all EM images. **f** Cholinergic terminals contained significantly higher immunogold signal than glutamatergic ones (*p* < 0.05) suggesting the presence of GABA in cholinergic terminals (median and interquartile ranges, Supplementary Note 8)

Then, we localised the elements of presynaptic GABAergic and cholinergic signalling machinery in these terminals. By crossing a zsGreen fluorescent reporter mouse-line with a vesicular GABA transporter (vGAT)-Cre mouse line and labelling the medial septum for ChAT, we found that all septo-hippocampal cholinergic cells are also vGAT positive (Fig. 2a, b, Supplementary Note 3). Hippocampal cholinergic terminals expressed the GABA-synthesising enzyme, glutamate decarboxylase 65 (GAD65) as well (Supplementary Note 3, Fig. 2c). In addition, at least 83% of cholinergic septo-hippocampal terminals were vGAT positive (Fig. 2d, Supplementary Note 3), whereas vesicular acetylcholine transporter (vAChT) was detected in 64% of the septo-hippocampal cholinergic terminals (Supplementary Note 4, 5). Finally, using postembedding immunogold staining, we showed that GABA is detectable in cholinergic terminals (Fig. 2e, f, Supplementary Note 3).

### Composite GABAergic–cholinergic postsynaptic responses

**Fig. 3:**
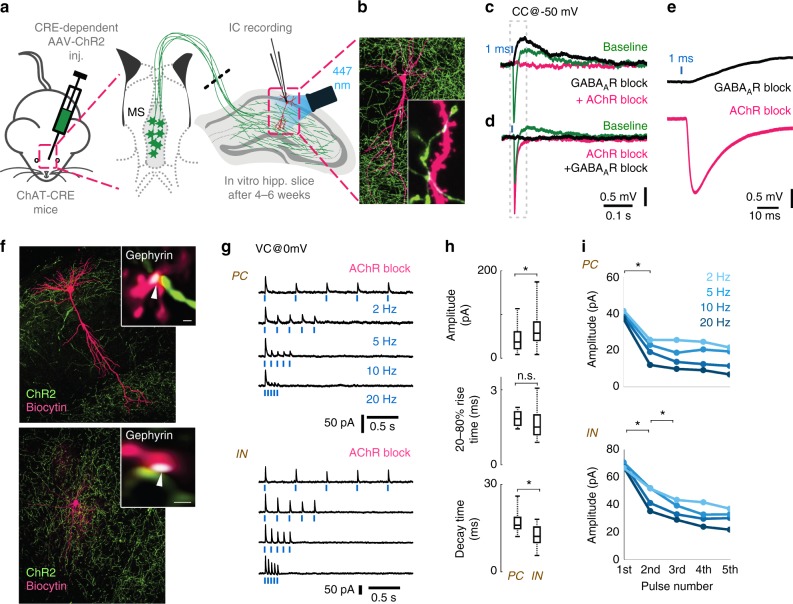
Optogenetic stimulation of cholinergic fibres elicits composite GABAergic and cholinergic postsynaptic responses. **a** Medial septum (MS) of ChAT-Cre mice were injected with Cre-dependent AAV containing Channelrhodopsin-2 (ChR2). Using whole-cell patch-clamp in horizontal hippocampal slices, we recorded voltage or current response from hippocampal neurons upon optical excitation of septo-hippocampal cholinergic fibres. NMDA and AMPA receptors were blocked with AP5 (50 µM) and NBQX (20 µM) in all experiments presented in Figs. 3 and 5. **b** Representative post-hoc visualised CA1 pyramidal cell (magenta) and the surrounding cholinergic fibres (green) with putative contacts (inset, white arrowheads). **c** Blue light pulses elicited a composite membrane potential response from str. lacunosum-moleculare inhibitory neurons (green, average of 50 stimulations). Inhibition of GABA_A_Rs (10 µM gabazine) abolishes the hyperpolarising component (nine from nine tested cells), resulting in a putative cholinergic excitatory potential (black). Inhibition of all AChRs (1 µM MLA, 1 µM DHβE, 10 µM atropine, four from four tested cells) blocks the remaining depolarising response (magenta). **d** Conversely, first blocking the AChRs, resulted in a putative GABAergic IPSP (magenta), which was blocked by GABA_A_Rs inhibitor gabazine (black). The increase of IPSP amplitude for AChR block is addressed in Fig. 5. **e** Magnified cholinergic EPSP (black) from **c**, and GABAergic IPSP from **d** (magenta) demonstrate their short latency (see also Supplementary Figure 3). **f** A representative recorded pyramidal cell (PC, top) and an inhibitory neuron (IN, bottom) post-hoc visualised in magenta and ChR2 positive cholinergic axons in green. Insets: Immunostaining for gephyrin (white) identify their putative synapses (white arrows). **g** We blocked both nicotinic and muscarinic AChRs (1 µM MLA, 1 µM DHβE, 10 µM atropine) and recorded inhibitory postsynaptic currents (IPSC) evoked by cholinergic fibre illumination. Traces show IPSC response of a PC and an IN to five light pulses (1 ms) at increasing stimulation frequencies (2, 5, 10, 20 Hz, cells were recorded in VC@0 mV). **h** Amplitude and decay time of unitary GABAergic IPSCs from pyramidal cells (*n* = 5) and inhibitory neurons (*n* = 16) were statistically different (*p* < 0.05), while their rise time was not different (median values, interquartile ranges and min/max values, see Supplementary Note 8). **i** Averages of IPSC amplitudes for the five pulses presented on **g** shows strong short-term depression (STD) of GABAergic transmission evoked by stimulating cholinergic fibres (for details see Supplementary Note 8 and Supplementary Figure 3)

We investigated the electrophysiological properties of these cholinergic responses in target neurons. We injected Cre-dependent, channelrhodopsin-2 (ChR2) expressing adeno-associated virus (AAV-ChR2) into the medial septum of ChAT-Cre knock-in mice (Fig. 3a, see Methods) and recorded hippocampal cells. AAV-ChR2 labelled only cholinergic cells in the septo-hippocampal pathway (see Supplementary Note 7) and cells of wild-type mice did not respond to illumination, proving that membrane potential responses in ChAT-Cre mice were caused only by cholinergic fibres. NMDA- and AMPA-type glutamate receptors were blocked to prevent polysynaptic recruitment of neuronal activity in all in vitro recordings presented in Fig. 3.

We recorded the membrane potential of inhibitory neurons in CA1 str. lacunosum-moleculare, because they are known to display cholinergic responses^26^. Cells were recorded using whole-cell patch-clamp in responses to 1 ms optical stimulation (Fig. 3a) that resulted in a composite membrane potential response: a GABA_A_ receptor-dependent hyperpolarization (peak @ 13.8 ms), and a slightly delayed (peak @ 92 ms) acetylcholine receptor-dependent depolarisation (Fig. 3c–e, Supplementary Figure 3A–C). Although these synaptically released transmitters may act on non-synaptic receptors as well, both responses had relatively short onset latency (2.8 and 7.4 ms, Supplementary Figure 3A–C) compared to typical non-synaptic transmission that has a typical evoked onset latency of about 60–160 ms^27^. Together with our anatomical data these suggest synaptic release and action of GABA and synaptic release and a very focal, synaptic and/or peri-synaptic action of acetylcholine. Synaptic spill-over of GABA and acetylcholine may act extrasynaptically as well.

Next, we blocked both nicotinic and muscarinic acetylcholine receptors (AChR) and recorded inhibitory postsynaptic currents (IPSC) on pyramidal cells (PCs) and interneurons (INs) after optical stimulation of cholinergic fibres (Fig. 3f–i). Single IPSC kinetics and short-term plasticity in PCs and INs were tested using five short light pulses at physiologically relevant firing rates (at 2, 5, 10 and 20 Hz) measured in vivo^28,29^. The amplitude of the evoked inhibitory currents (calculated for the first stimulus) was larger on INs than on PCs, but their rise time (20–80%) was not significantly different. IPSCs evoked in INs had a shorter decay time (Fig. 3h). The series of light pulses revealed strong short-term depression (STD) of inhibitory currents onto both PCs and INs (Fig. 3g, i). GABAergic STD was observed in every tested neuron and was accompanied by a decrease in transmission probability during the stimulation sequence (Supplementary Figure 3), suggesting a presynaptic mechanism for STD.

Calcium influx through ChR2 expressed on axon terminals can alter intrinsic short-term plasticity of the synapses^30^. To exclude this possibility, we used a digital micro-mirror device (DMD) inserted into the optical path of the microscope, to illuminate cholinergic axons running towards the recorded cell, but not the terminals (Supplementary Figure 3D). This resulted in STD similar to that recorded with optic fibre illumination (Supplementary Figure 3E–F), excluding the possibility of ChR2-mediated Ca^2+^ influx, as the reason for the observed STD. A series of stimuli could also decrease the driving force of chloride through GABA_A_Rs, resulting in apparent STD^31,32^. In this case, the putative site of STD would be postsynaptic instead of presynaptic. When we used a high Cl^−^ intracellular solution to prevent shifts in Cl^-^ reversal, a series of stimuli resulted in STD similar to that shown above (Supplementary Figure 3G and H).

### Cholinergic and GABAergic vesicle docking is restricted to synapses

**Fig. 4:**
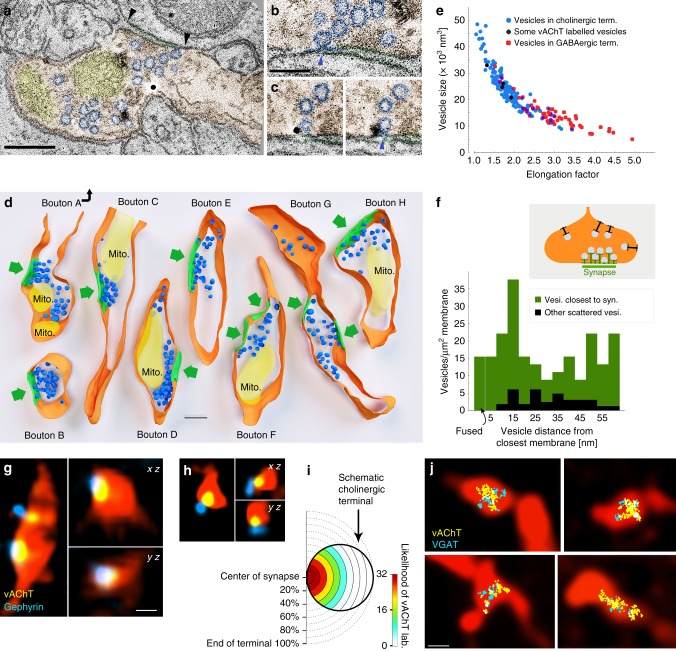
Separate vesicular release of acetylcholine and GABA from the same synaptic active zones. **a**–**c** About 0.5 nm-thick virtual sections from electron tomographic reconstructions of cholinergic terminals. Terminal is orange, mitochondria are yellow, synaptic vesicles are blue. Black arrowheads point to the edges of the synapse. Scale bar is 200 nm. **b** Active zone is enlarged from **a**. The omega-shaped vesicle is being fused with the terminal membrane (blue arrowhead). **c** Two serial virtual sections showing a portion of the active zone of another terminal. A fused synaptic vesicle (blue arrowhead) is labelled for vesicular acetylcholine transporter (black immunogold particle). Scale bar for **b** and **c** is 100 nm. **d** Three-dimensional models of reconstructed terminals. Terminal membranes are orange, mitochondria are yellow, synaptic vesicles are blue and synapses are green. Green arrows point to the synapses. Scale bar is 200 nm. The association of the vesicle pools to the active zones is evident. **e** Scatterplot shows the relationship of vesicle size (volume) and elongation factor in the sampled vesicles. Vesicles in the cholinergic terminals had a broader size-distribution than vesicles of purely GABAergic terminals, which were smaller and more elongated (Supplementary Note 4). Some vesicles (black markers) could be identified as vAChT-positive because immunogold particles clearly labelled them. **f** The density of the vesicles within a 60-nm-wide band from the membrane is plotted as a function of distance from the terminal membrane at synaptic (green) and extra-synaptic (black) areas. Vesicle density was 6.5 times higher (in the first 60 nm from the membrane) in the synaptic active zone than extrasynaptically (*n* = 183 vesicles from 8 terminals in 2 mice). Docked (closer to the membrane than 5 nm) or fused (undergoing exocytotic fusion) vesicles were absent extrasynaptically, but were present in the active zones. **g**, **h** vAChT labelling was confined to synaptic active zones in cholinergic fibres. CLSM images show terminals of virus-traced septo-hippocampal fibres (red pseudocolor) containing vAChT-positive vesicle pools (yellow), localised to the synaptic active zones that are identified by gephyrin labelling (blue). Scale bar is 500 nm for **g** and **h**. **i** Scale-free analysis confirms that the likelihood of vAChT labelling is the highest at the synaptic active zones (*n* = 32 synapses, 2 mice). **j** Correlated CLSM-STORM super-resolution microscopy images show that vAChT (yellow) and vGAT (cyan) vesicle pools are overlapping and are confined to small portions within AAV-eYFP virus-traced septo-hippocampal fibres (red pseudocolor)

Our physiological and anatomical data showed that cholinergic terminals establish synapses. Non-synaptic acetylcholine release, however, might still be possible, if cholinergic vesicles could be docked and fused outside of the synaptic active zones as well. Using advanced electron tomography tools, we were able to reconstruct cholinergic fibre segments, to localise vesicles with 1 nm resolution and to analyse the precise distribution of synaptic vesicles in hippocampal cholinergic terminals. We identified terminals using vAChT immunogold labelling and reconstructed them using electron tomography (Fig. 4a–d). Three-dimensional reconstruction revealed that synaptic vesicles clustered close to the active zones (Fig. 4d). We measured the distances of vesicles to the closest (synaptic or non-synaptic) cell membranes, and compared their density at different distance intervals from the membranes (Fig. 4f). The density of the vesicles within 60 nm from the membranes was 6.5 times higher in the synaptic active zone than extrasynaptically (Fig. 4d, f). We did not find any docked (<5 nm from the membrane) or fused (undergoing exocytotic fusion) vesicles non-synaptically, but we detected both of these at synapses (Fig. 4b, c, f). The distribution of vesicles in cholinergic terminals was similar to that found at other glutamatergic or GABAergic terminals, arguing against non-synaptic vesicular docking or release in cholinergic terminals.

Vesicular volume can correlate with its transmitter content^33^. Therefore, using electron tomography, we compared the vesicular morphology between cholinergic and GABAergic terminals (Fig. 4e). Vesicles of cholinergic terminals were significantly (*p* < 0.001), about 60% larger than those in GABAergic terminals (Fig. 4e, Supplementary Note 4) and their volumes were significantly more variable as well (*p* < 0.001). Smaller (putatively GABAergic) vesicles of cholinergic terminals were similar to those in purely GABAergic terminals (Fig. 4e, see Supplementary Note 4), suggesting an even larger difference between the two types of vesicles.

### Acetylcholine and GABA in different vesicles and same vesicle pool

To further confirm that the two transmitter systems use the same active zones, we used confocal fluorescent imaging in mouse hippocampus and found that vAChT-positive vesicles were concentrated at gephyrin-labelled synapses (Fig. 4g, h, Supplementary Note 5). Scale-free analysis confirmed that the likelihood of vAChT labelling was the highest at the gephyrin-labelled synaptic active zones (Fig. 4i, Supplementary Note 5).

Using super-resolution STORM imaging of vGAT and vAChT immunolabelling, combined with correlated fluorescent confocal laser-scanning microscopy (CLSM) of cholinergic fibres, we demonstrated that cholinergic–GABAergic vesicle pools were mixed and were confined to a small volume of the AAV-eYFP-labelled septo-hippocampal terminals (Fig. 4j, Supplementary Note 5).

Using isolated mouse cortical synaptic vesicles (neocortex and hippocampus, in ~4:1 ratio), we found that acetylcholine and GABA are packed into different vesicles (Supplementary Figure 4A, Supplementary Note 6). Both flow cytometry (Supplementary Figure 4B) and electron microscopy data (Supplementary Figure 4C, E) confirmed the purity of our sample (see also Supplementary Note 6 and Methods). All vesicles were labelled with synaptophysin (SYP). After quadruple-immunolabelling of the isolated synaptic vesicles, 29% were only SYP-positive, 45% were double labelled for vesicular glutamate transporter 1 and SYP, 14% were double labelled for vGAT and SYP, 11% were double labelled for vAChT and SYP. Only a negligible amount of vesicles (0.9%) were triple labelled with any combinations and only 0.14% of all vesicles were co-labelled for vAChT and vGAT, suggesting that vesicular transporters for GABA and acetylcholine are expressed by distinct vesicle populations in cortical samples (Supplementary Figure 4H; Supplementary Note 6).

### Different Ca-channel and mutual autoreceptor modulation

**Fig. 5:**
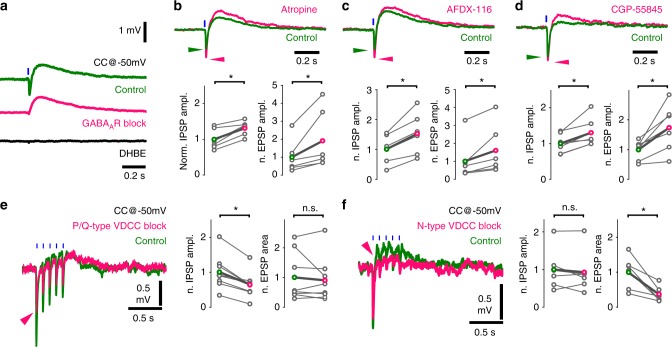
Co-transmission of GABA and acetylcholine is cross-regulated by autoreceptors and mediated by distinct calcium channels. **a** Optical stimulations of cholinergic fibres trigger composite membrane potential responses (average of 50 traces) of an inhibitory neuron recorded in str. lacunosum-moleculare (green). Cells were held at approximately −50 mV to record hyperpolarising and depolarising components concurrently. Early hyperpolarization was confirmed to be of GABAergic origin by bath application of the GABA_A_R blocker gabazine (magenta, 10 µM, which abolished quick hyperpolarization in 9 out of 9 tested cells), while the late depolarisation was confirmed to be cholinergic by the application of the alpha4-type nicotinic acetylcholine receptor blocker DHβE (black, 1 µM, which abolished depolarisation in 5 out of 5 tested cells). Cells with identifiable nicotinic responses were used to further investigate the properties of GABA and acetylcholine release from cholinergic fibres. **b** Increase in GABAergic response after AChR block (Fig. 3d) suggested that presynaptic muscarinic receptors cross-modulated GABA (and possibly ACh) release from cholinergic fibres. Therefore, putative presynaptic muscarinic receptors were selectively inhibited via bath application of atropine (*n* = 6), which led to a significant increase in amplitude of both the GABAergic hyperpolarization and nicotinic depolarisation (green, see Supplementary Note 8). **c** Bath application of the selective M2 antagonist AFDX-116 (10 µM, *n* = 6) resulted in changes similar to that of atropine application (for data see Supplementary Note 8). **d** Blocking GABA_B_Rs with CGP-55845 (1 µM, *n* = 7) also resulted in increased GABAergic and cholinergic responses suggesting a dual modulatory role (for data see Supplementary Note 8). **e**, **f** Testing roles of distinct voltage-dependent calcium channels (VDCC) in release of GABA and ACh . **e** Blockade of P/Q-type VDCC, using selective antagonist ω-agatoxin (1 µM, puff application), reliably decreased GABAergic response component but did not alter cholinergic component. At control conditions, 5 short (1 ms) light pulses at 10 Hz evoked a composite event of GABAergic hyperpolarization and cholinergic depolarisation (green, average of 20 traces). In response to ω-agatoxin application (magenta, *n* = 9), the GABAergic component (measured as IPSP amplitude) decreased robustly (magenta arrowhead) and the cholinergic component (measured as EPSP area) showed no change (for details see Supplementary Note 8). **f** Blockade of N-type VDCC, using selective blocker ω-conotoxin (1 µM), reliably reduced cholinergic response component (*n* = 6) but left GABAergic component unchanged (for details see Supplementary Note 4)

Our anatomical data indicated that acetylcholine and GABA are stored in, and thus likely released from different vesicles. First, we investigated presynaptic modulation of this vesicular release. Blocking AChRs increased the amplitude of GABAergic hyperpolarization (Fig. 3d), for which presynaptic muscarinic receptors may be responsible^26^. We held CA1 interneurons at −50 mV, to record GABAergic hyperpolarization and cholinergic depolarisation concurrently (Fig. 5a, NMDA- and AMPA-type glutamate receptors were blocked to prevent polysynaptic recruitment of neuronal activity in all in vitro recordings presented in Fig. 5). Muscarinic receptor blocker atropine (10 µM) significantly increased the amplitude of both GABAergic IPSPs and cholinergic EPSPs (Fig. 5b). By blocking M2-type AChRs, reported abundant in hippocampal cholinergic terminals^26,34^, we reproduced PSP increases evoked by atropine (Fig. 5c). This confirms the role of M2-type AChRs in regulating both acetylcholine and GABA release from cholinergic terminals.

Previously, we described the presence of presynaptic GABA_B_ autoreceptors in septal cholinergic cells^35^. To test their role in regulating synaptic co-transmission, we blocked GABA_B_ receptors. This led to a significant increase in the amplitude of both GABAergic IPSPs and cholinergic EPSPs (Fig. 5d). However, we did not see GABA_B_ receptor-dependent postsynaptic hyperpolarization in response to cholinergic fibre stimulation in our recordings. Overall, these experiments revealed that GABA and acetylcholine cross- and auto-regulate their co-transmission from cholinergic–GABAergic terminals, presynaptically.

Dynamics of vesicle release likely depends on the type of voltage-dependent calcium channels (VDCCs), which mediate Ca^2+^-triggered vesicle release^36^. However, different VDCCs in the same cell^20^ can be associated with distinct types of vesicles. Indeed, we found that selective blockade of P/Q-type calcium channels decreased GABAergic IPSPs significantly, but caused no change in the cholinergic components (Fig. 5e). Conversely, the cholinergic component was robustly decreased after the selective blockade of N-type calcium channels, while GABAergic IPSPs showed no significant change (Fig. 5f). Besides confirming the presence of different vesicular pools, this revealed different molecular pathways for their regulation during co-transmission.

Distinct vesicle pools of the same terminals can couple to different release mechanisms with different short-term plasticity^20^. Because GABAergic transmission always showed strong STD in cholinergic terminals (Fig. 3g, i), we examined, whether cholinergic responses display similar short-term dynamics. We recorded inhibitory neurons in the physiologically relevant current clamp (*I* = 0) situation, and repeatedly light-stimulated ChR2-expressing cholinergic axons (Supplementary Figure 3J). The net depolarisation for a series of five stimuli did not decrease. Also, the area under the voltage curve increased with frequency (Supplementary Figure 3J–K). In current clamp (more than in voltage clamp) even a non-depressing transmission could be seen as depressing due to reduction of driving force. Therefore, based on our current clamp measurements, it is highly unlikely that the cholinergic component would show such a strong STD as the GABAergic component.

### GABA release of cholinergic cells shape hippocampal states

**Fig. 6:**
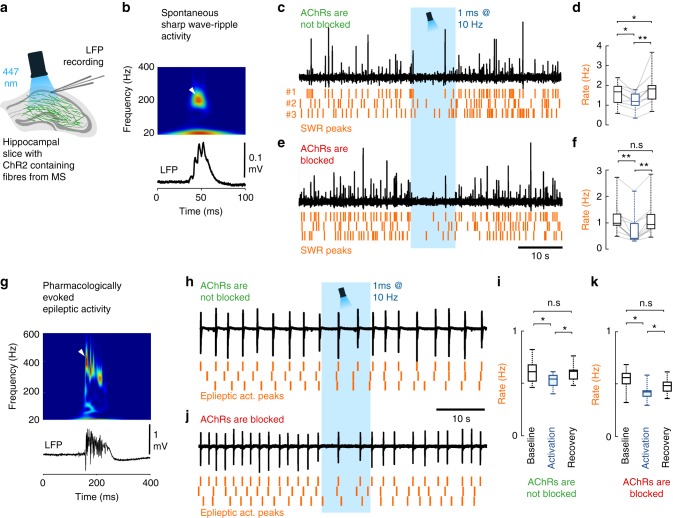
GABA release from cholinergic terminals controls healthy and pathological hippocampal network activity. **a** To understand the functional impact of GABA and acetylcholine co-transmission onto cortical network oscillations, local field potential (LFP) was recorded with standard patch pipettes from the CA1 area of thick hippocampal slices. These slices generated healthy and pathological in vivo like activity patterns. Cholinergic fibres expressing ChR2 were illuminated with blue light in the presence, or in the absence of AChR blockers parallel with LFP recordings. **b** A spontaneous sharp wave-ripple (SWR) LFP signature (bottom), and its wavelet transform (top) highlighting the characteristic ripple-band frequency component (~200 Hz, white arrowhead). **c**, **d** LFP recording from hippocampal CA1 in the absence of AChR blockers (*n* = 9). Orange raster indicate SWR peaks for three consecutive stimulation periods. Optical stimulation decreased SWR rate significantly (**d**), then SWR rate increased temporarily after the cessation of optical stimulation (median values, interquartile ranges and min/max values, for data see Supplementary Note 8). **e**, **f** After we blocked AChRs (1 µM MLA, 1 µM DHβE, 10 µM atropine), LFP recording from CA1 (*n* = 9) showed that GABA release from cholinergic fibres alone could decrease SWR rate even more effectively (**f**). There was no SWR rate increase after the cessation of optical stimulation (median values, interquartile ranges and min/max values, for data see Supplementary Note 8). **g** Epileptiform activity was evoked by elevating K^+^ concentration in the ACSF to 8.5 mM in slices. LFP (bottom) and its wavelet transform (top) shows the signature of a pharmacologically evoked epileptiform events. Note the robust differences in amplitude, length and frequency components compared to physiological SWR activity (Fig. 6b) that is consistent with literature data^41^. **h**, **i** Epileptic activity recorded in CA1 in the absence of AChR blockers (*n* = 7). Orange raster indicate detected epileptic activity peaks for three consecutive stimulation periods. Illumination of cholinergic fibres reduced the rate of epileptic events (**i**), which recovers after the cessation of optical stimulation (for data see Supplementary Note 8). **j**, **k** Epileptic activity recorded in CA1 in the presence of AChR blockers (*n* = 8). Even in the presence of AChR blockers, illumination of cholinergic fibres reduced the rate of epileptic discharges (**k**), which recovers after the stimulation (median values, interquartile ranges and min/max values, for data see Supplementary Note 8), suggesting a crucial role for GABA release in controlling epileptiform activity

Septal cholinergic neurons control hippocampal activity states^37,38^ and suppress in vivo sharp wave-ripples (SWR) in the hippocampus^39^. However, the effect of their hippocampal GABA release has not been investigated. We recorded spontaneous SWRs in hippocampal slices (Fig. 6, Methods). Illumination of control slices without ChR2 expression caused no change in SWR activity. In AAV-ChR2-infected ChAT-Cre mice, we found that without blocking cholinergic transmission, optical stimulation of cholinergic fibres decreased SWR rate significantly (Fig. 6c, d), followed by a transient increase in SWR rate after the cessation of optical stimuli. However, when cholinergic transmission was blocked, the same optical stimulation caused a similar decrease in SWR rate (Fig. 6e, f), with no rebound after stimulation. Although cholinergic fibres have been reported to be responsible for inhibiting SWRs^39^, here we demonstrated that GABA release from cholinergic synapses alone is sufficient to downregulate the rate of SWRs.

The GABA release from septo-hippocampal cholinergic fibres may also affect the occurrence of hippocampal epileptiform activity. Therefore, we examined the effect of GABA release from cholinergic axons in the in vitro high potassium epilepsy model (Fig. 6g–k)^40,41^. Optical stimulation of cholinergic fibres reduced the rate of epileptiform events (Fig. 6h, i) and a similar reduction was present, when AChRs were blocked (Fig. 6j, k). These results demonstrated that synaptic transmission of GABA from cholinergic fibres alone can effectively control hippocampal epileptiform activity.

## Discussion

Our results challenge the model of non-synaptic single transmitter release from cholinergic fibres in the hippocampus and demonstrate that all cholinergic terminals establish synapses, where GABA and acetylcholine are released into the same synaptic gap. Our results also show non-synaptic action of these synaptically released transmitters. We showed that hippocampal cholinergic terminals established effective GABAergic synapses as well. Cholinergic fibres are among the densest and most influential subcortical pathways in hippocampus; therefore, our findings suggest a fundamental change in our view of the regulation of hippocampal states. We also found that acetylcholine and GABA were not co-released but co-transmitted from the same synaptic active zone. We confirmed this by showing that (i) isolated cortical transmitter vesicles do not co-express vGAT and vAChT, (ii) they are preferentially modulated by distinct voltage-dependent calcium channels and (iii) electron tomography suggested differences in the volume of GABA and acetylcholine containing vesicles. This co-transmission (similar to that in retina^20^) may require nanoscale sub-synaptic organisation of presynaptic molecules, as proposed before^42,43^. Emphasising the functional role of GABA release, we also demonstrated that synaptic GABA release from cholinergic terminals alone can effectively suppress hippocampal sharp wave-ripples and per se can attenuate hippocampal epileptiform activity. These confirmed the functional importance of this GABAergic–cholinergic co-transmission in healthy and pathological states and led to a novel model of the septo-hippocampal cholinergic neurotransmission.

**Fig. 7:**
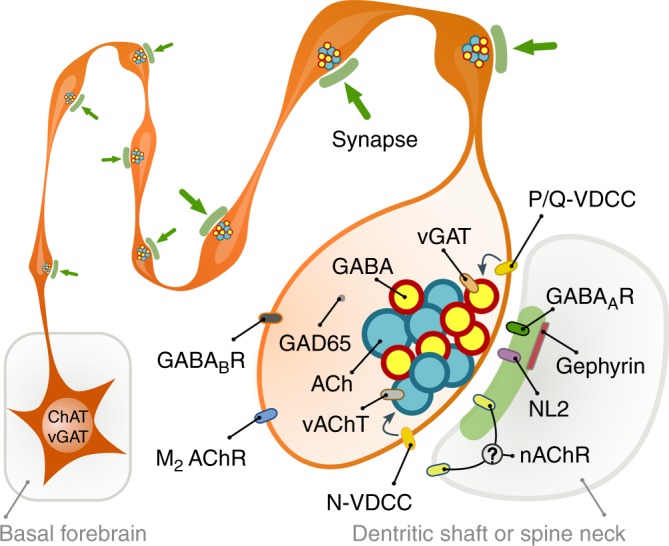
Illustration of cholinergic terminals and their synaptic architecture. Summary illustration of some of the findings. All cholinergic terminals establish synapses, they are fully equipped with GABAergic–cholinergic co-transmission signalling machinery. GABAergic and cholinergic vesicles are regulated by different VDCCs. Although postsynaptic cholinergic receptor distribution cannot be investigated, their response latencies (that are at least and order of magnitude faster that typical non-synaptic transmission) suggest a focal, intra- and/or peri-synaptic localisation, while GABA_A_ receptors are detected intra-synaptically. Synapses are established on both dendritic shafts and spines in hippocampus

For decades, the predominant form of cholinergic communication was thought to be a form of non-synaptic release^8–10,12,44^, which was seemingly supported by studies showing cholinergic fibres with few synapses (for details see Supplementary Discussion). Originally, the presence of extra-synaptic acetylcholine receptors and micro-dialysis experiments seemed to support a non-synaptic release hypothesis; however, later, sensitive microelectrodes showed faster changes in extracellular acetylcholine levels (Supplementary Discussion). In addition, basal forebrain cholinergic neurons were also shown to respond to reward and punishment with extremely high speed and precision^14^, and recent data suggested that cholinergic cells may regulate cortical information processing with a remarkable, millisecond-scale temporal precision^15,17,45–48^. These data urged the re-examination of whether acetylcholine release is synaptic^49^. Using electron tomography and direct labelling of synapses for neuroligin 2, we showed that, all terminals of the dense meshwork of hippocampal cholinergic fibres established (one or more) synapses and no docked or fused vesicles were detectable non-synaptically. Most of these synapses were missed previously, probably, because of their weak membrane thickening and narrower synaptic gap. Furthermore, we found that vAChT-labelled vesicle pools associated only with synaptic active zones (Fig. 7).

Non-synaptic transmission has a typical delayed onset latency of about 60–160 ms^27^, while synaptic onset latencies are typically more than an order of magnitude faster. For instance, the average latency with optogenetic stimulation can be as long as 6.4^50^ or 5.6 ms^51^ for monosynaptic glutamate transmission. Our data show the release of acetylcholine into the synaptic active zone and a 7.4 ms onset cholinergic response latency. These response latencies suggest a focal, intra- and/or peri-synaptic localisation of nicotinic receptors, while GABA_A_ receptors are detected intra-synaptically. However, future work will be required to resolve the contribution of intra- and/or peri-synaptic receptors to the cholinergic response, because measured, relatively fast latency alone cannot reveal the distribution of postsynaptic receptors around synapses. Although none of our data suggests “volume transmission” of acetylcholine or GABA, they may still act on extra-synaptic receptors as well, in a “volume transmission” fashion. In fact, the most likely resolution for the mismatch between our and the classic non-synaptic volume transmission hypothesis is a strong “spill-over” of transmitters from these frequently occurring synapses that would allow transmitters to reach extra-synaptic receptors as well. In fact, the much larger cholinergic vesicles that we found could contain more acetylcholine to counteract its effective extra-synaptic removal by extracellular acetylcholine esterase.

Co-transmission can significantly increase the efficacy of information transfer^52–54^. Using purified vesicle preparation, we showed that GABA and acetylcholine are not “co-released” from the same vesicles, but “co-transmitted” from separate vesicles, sequentially inhibiting and exciting hippocampal neurons. To unlock the full potential of co-transmission, these coexisting vesicle pools need complex regulation. Indeed, we showed that the release of GABAergic vesicles are preferentially regulated by P/Q-type calcium channels, while the cholinergic ones are more affected by N-type calcium channels in the same synapses (Fig. 7), similar to that of retinal co-transmission of GABA and acetylcholine^20^, therefore, an activity-dependent segregation in their release is also possible. Under special circumstances one of these transmitters may even be depleted earlier (GABAergic transmission shows strong STD). Differential calcium channel regulation may be achieved by sub-synaptic nanoscale organisation of presynaptic molecules^42,43^, while the presence of two different kinds of presynaptic channel “slots” have already been suggested at excitatory synapses: one accepting N-type channels but rejecting P/Q-type (N-specific) and the other preferring P/Q-type but also accepting N-type (PQ preferring^53^,).

Negative feedback via presynaptic autoreceptors could provide a further level of control (Fig. 7). Previously, we demonstrated that septo-hippocampal cholinergic neurons express GABA_B_-receptors^35^, while the expression of muscarinic M2 receptors was also described in these cells^34^. Indeed, we confirmed that blocking either M2- or GABA_B_-receptors increased the release of both acetylcholine and GABA, suggesting the presence of tonically active autoreceptor regulation of these transmitters, presynaptically. In addition, our results, verified by a series of control experiments, suggested that GABAergic STD is an intrinsic property of synapses established by cholinergic fibres in the mouse hippocampus.

The timing of this co-transmission seems crucially important in hippocampal Schaffer collateral (SC) to CA1 synaptic plasticity. If the cholinergic input was activated 100 or 10 ms before SC stimulation, it resulted in ionotropic AChR-dependent long-term potentiation (LTP) or short-term depression, respectively, while delaying cholinergic stimulation until 10 ms after SC stimulation resulted in metabotropic AChR-dependent LTP^17^. Here, we found that the GABAergic and cholinergic components of this co-transmission reach their IPSP and EPSP peak about 13.8 and 92 ms after stimulation, respectively, which suggest that synaptic plasticity may depend on the coincidence of SC stimulation with the GABAergic or cholinergic component of PSPs from these basal forebrain fibres. Nevertheless, the GABAergic component seems to have its own inhibitory role in the target network, making basal forebrain cholinergic fibres an unorthodox but effective source of GABAergic inhibitory control of the hippocampus, as suggested by our demonstration of its effect on network dynamics.

Basal forebrain cholinergic cells play a pivotal role in transforming activity states in the hippocampus^37^. High cholinergic cell activity is associated with theta oscillation and the fast, yet unstable storage of external information in the hippocampus, while low cholinergic activity accompanies sharp wave-ripple (SWR) activity^55^, which seems important for the consolidation and relocation of unstable memory traces from the hippocampus to the neocortex^56,57^. Classic theories of cholinergic modulation presume that diffusely released acetylcholine would slowly retune cortical network activity, enabling the appearance of distinct network dynamics^8,58,59^. Indeed, a recent study demonstrated that medial septal cholinergic cells suppress SWR activity in vivo^39^. The authors suspected an M2 cholinergic receptor-mediated suppression of GABAergic interneurons. We re-examined that hypothesis and confirmed that cholinergic fibre activation indeed suppressed SWRs; however, we also showed that GABA (without acetylcholine) release from cholinergic fibres alone was able to achieve that. This GABAergic inhibition could lower the probability of concurrently active pyramidal cells in a given time-window, which is required for stochastic SWR initiation^60^. Blocking all GABA receptors would have eliminated SWRs^61^, therefore the effect of acetylcholine release alone, without the contribution of GABA co-transmission could not be addressed. The presence of a transient increase in SWR rate after the cessation of optical stimuli, might reflect longer-lasting changes in network or cell excitability, mediated by “synaptic spill-over” of acetylcholine, while the effect of GABA release from smaller vesicles is likely confined to synaptic (and presynaptic) receptors. However, this transient effect was not reported in vivo^39^, therefore, it could have been the result of a relatively low acetylcholine esterase activity in our dual-superfusion system.

Degeneration of the cholinergic system is a typical characteristic of Alzheimer’s disease pathology^7^, and these patients often develop epileptic seizures^62^. Indeed, selective lesion of medial septal cholinergic projection increases seizure incidence in the hippocampus^63–65^, while REM-sleep, which is associated with enhanced cholinergic cell activity^29^, has a suppressing effect on epileptic seizures^66,67^. The mechanisms, however, were unclear, because several cholinergic agonists were shown to trigger epileptic discharges^68^. Therefore, we hypothesise that degeneration of basal forebrain cholinergic cells leads to epileptiform activity primarily, because it deprives the hippocampus of a massive GABAergic input. Indeed, our results supported this idea, because GABA release from basal forebrain cholinergic fibres alone was sufficient to decrease the occurrence of epileptiform activity in the hippocampus.

Our results, showing a tightly regulated, effective, synaptic GABA co-transmission from hippocampal cholinergic fibres urge the re-interpretation of previous models and can lead to alternative pharmacotherapies to treat Alzheimer’s disease-related loss of cholinergic innervation.

## Methods

### Animals and surgery

A total of 21 (25–80 days old) male C57BL/6J mice, 51 (30–200 days old) ChAT-IRES-Cre mice from either sex (Jackson Laboratory, RRID: IMSR_JAX:006410, postnatal day 30–200) and 4 (30–50 days old) VGAT-IRES-Cre/Gt(ROSA)26Sor_CAG/ZsGreen1 mice were used in the present study. All experiments were performed in accordance with the Institutional Ethical Codex, Hungarian Act of Animal Care and Experimentation (1998, XXVIII, section 243/1998) and the European Union guidelines (directive 2010/63/EU), and with the approval of the Institutional Animal Care and Use Committee of the Institute of Experimental Medicine of the Hungarian Academy of Sciences. All efforts were made to minimise potential pain or suffering and to reduce the number of animals used.

ChAT-Cre mice were anaesthetised with isoflurane followed by an intraperitoneal injection of an anaesthetic mixture (containing 8.3 mg/ml ketamine, 1.7 mg/ml xylazine-hydrochloride in 0.9 % saline, 10 ml/kg bodyweight) and then were mounted in a stereotaxic frame. For selective labelling of septo-hippocampal cholinergic axons, we injected 30–60 nl AAV2/5-EF1a-DIO-eYFP (UNC Vector Core) or AAV2/5-EF1a-DIO-ChR2(H134R)-eYFP-WPRE-hGH (plasmid: Addgene 20298, Penn Vector Core) into the medial septal area. The coordinates for the injection were based on the atlas by Paxinos, G and Franklin, (2012)^69^: 1 mm anterior from the bregma, in the midline, and 4.3 mm below the level of the horizontal plane defined by the bregma and the lambda (zero level). For a control experiment, 100 nl injections of the same virus were delivered into the hippocampus (both hemispheres) of a ChAT-Cre mouse (coordinates: −2.7 mm from the bregma, ±2.5 mm from the midline, 2.5 mm from the zero level and −3.1 from the bregma, ±3 mm from the midline and 3 mm from the zero level). For the injections, we used a Nanoject 2010 precision microinjector pump (WPI, Sarasota, FL 34240). We used borosilicate micropipettes (Drummond, Broomall, PA) for the injections with tips broken to 40–50 µm. After the surgeries, the animals received 0.5–0.7 ml saline for rehydration and 0.03–0.05 mg/kg meloxicam as nonsteroidal anti-inflammatory drug (Metacam, Boehringer Ingelheim, Germany) intraperitoneally to support recovery, and we placed them into separate cages to survive for at least three weeks before decapitations or perfusions.

### Slice preparation and recording conditions

After 3–6 weeks following injection (to reach an appropriate expression level in long-range projecting axons) horizontal slices were prepared. In all cases, mice were decapitated under deep isoflurane anaesthesia. The brain was removed into ice-cold cutting solution, which had been bubbled with 95% O_2_–5% CO_2_ (carbogen gas) for at least 15 min before use. The cutting solution contained the following (in mM): 205 sucrose, 2.5 KCl, 26 NaHCO_3_, 0.5 CaCl_2_, 5 MgCl_2_, 1.25 NaH_2_PO_4_, 10 glucose, saturated with 95% O_2_–5% CO_2_. Horizontal hippocampal slices of 300 µm or 450 µm thicknesses (for whole-cell and LFP recordings, respectively) were cut using a vibratome (Leica VT1000S). After acute slice preparation the slices were placed into an interface-type holding chamber for recovery. This chamber contained standard artificial cerebrospinal fluid (ACSF) at 35 °C that gradually cooled down to room temperature. The ACSF had the following composition (in mM): 126 NaCl, 2.5 KCl, 26 NaHCO_3_, 2 CaCl_2_, 2 MgCl_2_, 1.25 NaH_2_PO_4_, 10 glucose, saturated with 95% O_2_–5% CO_2_. After incubation for at least 1.5 h, slices were transferred individually into a submerged-style recording chamber equipped with a single superfusion system. In case of LFP recording of hippocampal network activities, a double superfusion system was used for improved slices maintenance conditions^60,70^. In the latter design, the slices were placed on a metal mesh and two separate fluid inlets allowed ACSF to flow both above and below the slices with a rate of 3–5 ml/min for each flow channel at 30–32 °C (Supertech Instruments; www.super-tech.eu). The composition of the modified ACSF (mACSF) used in experiment presented in Fig. 6g–k was the following (in mM): 126 NaCl, 3.5 KCl, 26 NaHCO_3_, 1.6 CaCl_2_, 1.2 MgCl_2_, 1.25 NaH_2_PO_4_, 10 glucose saturated with 95% O_2_–5% CO_2_. Epileptic events were evoked by further increasing the potassium concentration from 3.5 to 8.5 mM after 15 min spent in mACSF in the recording chamber. Slices were visualised with an upright microscope (Nikon Eclipse FN1 or Olympus BX61WI) with infrared-differential interference contrast optics. Standard patch electrodes were used in all recording configurations (i.e., in whole-cell patch-clamp and field potential recordings). Pipette resistances were 3–6 MΩ when filled either with the intracellular solution or with ACSF. ACSF filled pipettes placed into CA1 pyramidal layer were used for local field potential (LFP) recordings. The composition of the intracellular pipette solution was the following (in mM): 110 K-gluconate, 4 NaCl, 20 HEPES, 0.1 EGTA, 10 phosphocreatine, 2 ATP, 0.3 GTP, 3 mg/ml biocytin adjusted to pH 7.3–7.35 using KOH (285–295 mOsm/L). Where indicated, high-chloride containing intracellular solution was used: 54 K-gluconate, 4 NaCl, 56 KCl, 20 HEPES, 0.1 EGTA, 10 phosphocreatine, 2 ATP, 0.3 GTP, 3 mg/ml biocytin adjusted to pH 7.3–7.35 using KOH (285–295 mOsm/L). Whole-cell series resistance was in the range of 5–15 MΩ. Series resistance was not compensated but was frequently monitored, and cells where the values changed more than 25% during recording were discarded from further analysis. Voltage measurements were not corrected for the liquid junction potential.

### Drugs

To avoid polysynaptic effects in response to optical stimulation of cholinergic fibres, glutamatergic excitatory currents were blocked by 20 μM NBQX and 50 μM AP5 in all experiments presented on Figs. 3, 5 and Supplementary Figure 3. The following drug concentrations were used in the specified experiments (wash application): 20 µM NBQX, 50 µM AP5, 10 µM atropine, 1 µM MLA, 1 µM DHBE, 10 µM gabazine, 1 µM CGP-55845. For puff application of ω-conotoxin (1 µM) and ω-agatoxin (1 µM), a glass capillary was placed adjacent to the recorded cell. These drugs were injected by mouth-applied pressure for 1–2 min during the stimulation protocol following the control period. Drugs were dissolved in HEPES-based buffer with a composition in mM: 126 NaCl, 10 glucose, 2.5 KCl, 1.25 NaH_2_PO_4_, 2 CaCl_2_, 2 MgCl_2_, 26 HEPES, pH 7.3. Gabazine (SR 95531 hydrobromide), CGP-55845, MLA (Methyllycaconitine citrate), DhβE (dihydro-β-erythroidine hydrobromide) and AFDX-116 were purchased from Tocris Bioscience, ω-conotoxin and ω-agatoxin were purchased from Almone labs, and NBQX (2,3-Dioxo-6-nitro-1,2,3,4-tetrahydrobenzo[f]quinoxaline-7-sulfonamide) and AP5 were purchased from Hello Bio. All other salts and drugs were obtained from Sigma-Aldrich or Molar Chemicals KFT.

### Anatomical identification

The recorded cells were filled with biocytin. After the recording, the slices were fixed in 4% paraformaldehyde in 0.1 M phosphate buffer (PB; pH 7.4) for at least 3 h, followed by washout with PB several times. Then, sections were blocked with normal goat serum (NGS; 10%) diluted in Tris-buffered saline (pH 7.4), followed by incubations in Alexa Fluor 594-conjugated streptavidin (1:1000; Invitrogen). Sections were then mounted on slides in Vectashield (Vector Laboratories) and were morphologically identified on the basis of their location, dendritic and axonal arborisation.

### Optogenetic illumination

For illumination, we used a blue laser diode (447 nm, Roithner LaserTechnik GmbH) attached to a single optic fibre (Thorlabs) positioned above the hippocampal slice. In all cases, 1 ms pulse length was used. In case of structured illumination (Supplementary Figure 3), the same laser was used with a digital micro-mirror device (Polygon400, Mightex Systems, Toronto, Canada) integrated into the optical path of the microscope. We have used Polygon400 for the illumination of the slice at a remote location (~5–600 µm), to avoid direct illumination of ChR2-expressing terminals on the recorded cell.

### Electrophysiological recordings and analysis

Both whole-cell and LFP recordings were performed with a Multiclamp 700A or 700B amplifier (Molecular Devices) and were low-pass filtered at 3 kHz using the built-in Bessel filter of the amplifier. Data were digitised at 10 kHz with a PCI-6042E board (National Instruments) and recorded with EVAN 1.3 software (courtesy of Prof. I. Mody, University of California Los Angeles, Los Angeles, CA). All data were analysed off-line using custom-made programmes written in MATLAB 8.5.0, Python 2.7 by D.S. and Delphi 6.0 by A.I.G.

The kinetic properties (20–80% rise times, decay time constant, latency) were calculated on stimulus triggered averaged events. Fitting of a single exponential function to the decaying phase of averaged responses and statistical analyses were performed in OriginPro, version 9.2.214 (OriginLab Corporation, Northampton, MA, USA). Latency from stimulation start to PSP onset was calculated as the time when the signal crossed three times standard deviation of baseline. Transmission probability was defined, as the probability of detectable IPSC in response to the optical stimulation. For characterising short-term plasticity, each stimulation frequency was repeated 20 times with an interval of 3 s. To record in parallel the changes in cholinergic EPSP and GABAergic IPSPs (for the drug applications presented on Fig. 5) cells were measured at −50 mV in current clamp mode. Since the amplitude of both PSPs increased, qualitative changes in amplitude could be measured, however in this way, we may have underestimated the changes in amplitude due to conductances overlapping in time and acting in the opposite directions. In the case of VDCC blocker application, we used five pulses to evoke larger cholinergic responses. We quantified EPSP area changes for ω-agatoxin and ω-conotoxin application after low-pass filtering (at 2 Hz) the response (e.g. subtracting GABAergic component).

Figure 5 data were normalised for visualisation (all values were divided by the mean of the control sample)^71^. LFP signals were filtered with a two way RC filter to preserve phase. SWRs were pre-detected on 30 Hz low-pass-filtered field recordings using a threshold value of four times the SD of the signal. The pre-detected SWRs were then redetected using a programme that detected SWR peaks and eliminated recording artefacts similar to SWRs. Epileptic event peaks were detected similarly. Event rates were calculated with equal time periods for control, stimulation and recovery phases. All automatic detection steps were manually supervised.

### Perfusion

For perfusion, mice were deeply anaesthetised as above. Mice for electron microscopical reconstruction of axonal segments and immunofluorescent labellings were perfused transcardially first with 0.9% NaCl in 0.1 M phosphate buffer (PBS) solution for 2 min followed by 4% paraformaldehyde (PFA) in 0.1 M phosphate buffer (pH = 7.4; PB) for 40 min and finally with PBS for 10 min. In the case of one WT mouse for reconstruction, the fixative also contained 0.5% glutaraldehyde. Two WT mice for postembedding GABA-labelling and electron tomography were perfused first with PBS for 2 min followed by a fixative containing 2% PFA and 2% glutaraldehyde in 0.1 M sodium acetate buffer (pH 6.5) for 2 min, and then by 2% PFA/2% glutaraldehyde in 0.1 M sodium borate buffer (pH 8.5) for 1 h^72^. Brains were removed from the skull and postfixed overnight at 4 °C in 2% PFA/2% glutaraldehyde in 0.1 M sodium borate buffer (pH 8.5). Mice for electron microscopical or immunfluorescent detection of gephyrin and GABA_A_ receptors were perfused transcardially with ice-cold, oxygenated artificial cerebrospinal fluid [containing (mM) NaCl 125, KCl 2.5, CaCl_2_ 2.5, MgCl_2_ 2, NaHCO_3_ 26, NaH_2_PO_4_ 1.25, glucose 25^73^]. Then the brain was removed from the skull and cut in blocks containing the hippocampal formation (separated hemispheres) and the medial septal area. The blocks were incubated in a fixative containing 4% PFA and 0.2% glutaraldehyde in PB for 90 min at 4 °C and then embedded in 2% agarose for sectioning. Coronal sections were cut on a Leica VT1200S vibratome at 50 µm. All series of sections were rinsed in PB, cryoprotected sequentially in PB containing 10 and 30% sucrose, frozen in liquid nitrogen and stored at –70 °C until further processing.

### Antibodies

We have summarised the primary antibodies used, their concentrations and information on their specificity in Supplementary Table 1. The secondary antibodies (Supplementary Table 2) were extensively tested for possible cross-reactivity with the other secondary or primary antibodies, and possible tissue labelling without primary antibodies was also tested to exclude auto-fluorescence or specific background labelling by the secondary antibodies. No specific-like staining was observed under these control conditions.

### Single- and double-labelling pre-embedding immunoelectron microscopy

Sections were freeze-thawed two times over liquid nitrogen and washed in PB. Sections for gephyrin immunolabelling were treated with 1% sodium borohydride in PB for 10 min. For detection of GABA_A_ receptors, the sections were incubated in 0.2 M HCl solution containing 2 mg/ml pepsin (Dako) at 37 °C for 2–4 min. After extensive washes in PB and 0.05 M Tris-buffered saline (pH 7.4; TBS) sections were blocked in 1% human serum albumin (HSA; Sigma-Aldrich) in TBS. Then, they were incubated in a mixture of primary antibodies (Supplementary Table 1) diluted in TBS containing 0.05% sodium azide for 2–3 days. After repeated washes in TBS, the sections were incubated in blocking solution (Gel-BS) containing 0.2% cold water fish skin gelatine and 0.5% HSA in TBS for 1 h. Next, sections were incubated in gold-conjugated and biotinylated secondary antibodies (Supplementary Table 1) diluted in Gel-BS overnight. After extensive washes in TBS the sections were treated with 2% glutaraldehyde in 0.1 M PB for 15 min to fix the gold particles into the tissue. This was followed by incubation in avidin–biotinylated horseradish peroxidase complex (Elite ABC; 1:300; Vector Laboratories) diluted in TBS for 3 h at room temperature or overnight at 4 °C. The immunoperoxidase reaction was developed using 3,3-diaminobenzidine (DAB; Sigma-Aldrich) as chromogen. To enlarge immunogold particles, sections were incubated in silver enhancement solution (SE-EM; Aurion) for 40–60 min at room temperature. The sections were then treated with 1% (for electron tomography) or 0.5% OsO_4_ in 0.1 M PB, at room temperature (for electron tomography) or on ice, dehydrated in ascending alcohol series and in acetonitrile and embedded in Durcupan (ACM; Fluka). During dehydration, the sections were treated with 1% uranyl acetate in 70% ethanol for 20 min. For electron microscopic analysis, tissue samples from the CA1 area of dorsal hippocampus/somatosensory cortex (S1) were glued onto Durcupan blocks. Consecutive 70 nm-thick (for conventional electron microscopic analysis) or 150 nm-thick (for electron tomography) sections were cut using an ultramicrotome (Leica EM UC6) and picked upon Formvar-coated single-slot grids. Ultrathin sections for conventional electron microscopic analysis were counterstained with lead citrate (Ultrostain 2, Leica) and examined in a Hitachi 7100 electron microscope equipped with a Veleta CCD camera (Olympus Soft Imaging Solutions, Germany). 150 nm-thick electron tomography sections were examined in FEI Tecnai Spirit G2 BioTwin TEM equipped with an Eagle 4k HS camera.

### Combination of pre- and postembedding immunocytochemistry

vAChT was visualised using the pre-embedding gold method as described above. Alternate serial 70 nm-thick sections were mounted on copper and nickel grids (5–6 sections/grid). Postembedding GABA immunostaining was carried out on nickel grids according to a modified protocol^74^. Incubations of sections were performed on droplets of solutions in humid Petri dishes in the following order: 0.5% periodic acid (H_5_IO_6_) for 5 min at room temperature; three times at 2 min wash in distilled water; 3 min in TBS; 15 min in 1% ovalbumin dissolved in TBS at 37 °C; 8 min in TBS, 90 min rabbit anti-GABA antiserum (1:10,000 in TBS) at 37 °C; two times at 10 min TBS; 10 min in TBS containing 1% BSA and 0.05% Tween 20; 90 min at room temperature in 10 nm colloidal gold-conjugated goat anti-rabbit IgG (BBI solutions; 1:1000 in the same solution as before); three times at 5 min wash in distilled water; 20 min in 10% saturated uranyl acetate; 4 wash in distilled water; staining with lead citrate; wash in distilled water. The etching procedure of postembedding GABA immunostaining removes the silver precipitate of the pre-embedding vAChT labelling (Fig. 2e); therefore, only every second electron microscope grid was reacted for GABA and the analysis of vACHT-positive terminals were carried out using the so called mirror technique. Sections on copper grids adjacent to the first sections on nickel grids were systemically scanned for pre-embedding immunogold-labelled vAChT-positive terminals. These terminals were identified in the next, GABA-labelled section, followed in consecutive serial sections and digital images were taken at 50,000 times magnification in each serial section. Postembedding immunogold particles were counted on these GABA-labelled terminals and the examined surface area was measured using the Reconstruct software^75^. For comparison, we have also measured the immunogold densities of putative glutamatergic terminals using the same method, in the same series of images. Terminals forming type-I (asymmetric) synapses were considered glutamatergic. Although periodic acid-etching of the sections is known to remove pre-embedding antibodies together with immunogold and silver precipitation, we confirmed this again in a control experiment because, here, both the pre-embedding labelling and the postembedding GABA staining was performed using rabbit antibodies. In these control experiments, the postembedding GABA immunostaining was carried out without the primary anti-GABA antibody, in which case practically no postembedding gold particles were detected, confirming the specificity of the method.

### Electron microscopy analysis of synapses of cholinergic fibres

For evaluation of the gephyrin and GABA_A_ receptor content at synapses of cholinergic axons, we performed pre-embedding double labelling. vAChT (in wild-type mice) or eYFP (in ChAT-Cre mice, see above) was labelled with DAB, while gephyrin or GABA_A_ γ2 subunit was labelled with immunogold (see above, Supplementary Tables 1–2). Electron microscopic serial sections were systemically scanned for synapses of DAB-labelled terminals. Parallel appositions between the membranes of a DAB-containing terminal and a putative postsynaptic target were regarded as a synapse if they displayed widening of the extracellular space at the presumptive synaptic cleft and clustered synaptic vesicles in the terminal. Synapses found were followed and photographed at 30,000 magnification in every section, where they were present throughout the series: thus they were fully reconstructed. Since the background labelling (measured in putative glutamatergic synapses in the same series of sections) was negligible, synapses containing at least one gold particle were regarded as positive.

### 3D reconstruction of axonal segments

Cholinergic axonal segments (*n* = 17) were reconstructed using consecutive serial 70 nm-thick sections double labelled for choline acetyltransferase (ChAT; DAB) and neuroligin 2 (NL2; immunogold) in wild-type mice (*n* = 2) or eYFP (DAB) and gephyrin (immunogold) in ChAT-Cre animals, in which the medial septum was injected with Cre-dependent eGFP-containing virus (*n* = 2). CB_1_-positive axons were reconstructed from a series of 70 nm-thick sections double labelled for CB_1_ (DAB) and NL2 (immunogold). DAB-containing axons were followed in consecutive serial sections and digital images were taken at 30,000 times magnification in each serial section. Three-dimensional reconstructions of plasma membranes, mitochondria, putative synapses of DAB-containing axons and postsynaptic gold particles were made using the Reconstruct software^75^. For preparing figures of reconstructed axons and measuring their 3D length, Blender software (www.blender.org) was used. Postsynaptic targets of cholinergic axons were classified as described earlier^76^. Briefly, spines were recognised by their small size and specific morphology. In CA1 str. oriens and radiatum, dendrites that have spines and do not receive type-I (asymmetric) inputs on their shafts are known to be pyramidal cells^77^, whereas dendrites receiving type-I synapses on their shafts are interneurons^78^. The robustness of this classification method was reconfirmed recently^76^. Because in str. lacunosum-moleculare the shafts of pyramidal dendrites may receive type-1 inputs^77^ they were not distinguished from interneuron dendritic shafts. Also, the dendritic shafts in somatosensory cortex S1 could not be identified based on morphological features.

### Immunofluorescent labelling and confocal laser-scanning microscopy

Before the immunofluorescent staining, the 50 µm thick sections were washed in PB and Tris-buffered saline (TBS). This was followed by blocking for 1 h in 1% human serum albumin (HSA) and 0.1% Triton X-100 dissolved in TBS. After this, sections were incubated in mixtures of primary antibodies overnight at room temperature. After incubation, sections were washed in TBS, and were incubated overnight at 4 °C in the mixture of, all diluted in TBS. Secondary antibody incubation was followed by washes in TBS, PB, the sections were mounted on glass slides and coverslipped with Aqua-Poly/Mount (Polysciences). Immunofluorescence was analysed using a Nikon Eclipse Ti-E inverted microscope (Nikon Instruments Europe B.V., Amsterdam, The Netherlands), with a CFI Plan Apochromat VC 60XH oil immersion objective (numerical aperture: 1.4) and an A1R laser confocal system. We used 405, 488, 561 and 647 nm lasers (CVI Melles Griot), and scanning was done in line serial mode. Image stacks were obtained with NIS-Elements AR software, and deconvolved using Huygens Professional software (www.svi.nl).

### STORM super-resolution microscopy

Before the immunofluorescent staining for the super-resolution experiments, the 20 µm thick sections were washed in PB and TBS. This was followed by blocking for 1 h in 1% HSA dissolved in TBS. After this, sections were incubated in mixtures of primary antibodies; overnight at room temperature. After incubation, sections were washed in TBS, and sections were incubated overnight at 4 °C in the mixtures of secondary antibodies. Secondary antibody incubation was followed by washing in TBS, PB; then hippocampi were cut out with scalpels in buffer, sections were dried on clean coverslips, and stored for maximum 3 weeks at room temperature in a dry environment before imaging. 3D direct-STORM (direct Stochastic Optical Reconstruction Microscopy) acquisition protocol was used as described before^79^. The imaging setup was built around a Nikon Ti-E inverted microscope equipped with a Perfect Focus System, with an Andor iXon Ultra 897 EMCCD camera, a C2 confocal head, 405, 488 and 561 nm lasers (Melles Griot 56RCS/S2780, Coherent Sapphire), and a high power 647 nm laser (300 mW, MPB Communications VFL-P-300-647). A high NA ×100 oil immersion objective (Nikon CFI SR Apochromat TIRF ×100 oil, 1.49 NA) was used for imaging. During STORM acquisition the emitted light was let through 582/636 nm and 670/760 nm bandpass filters to reach the detector for the 561 and 647 channels, respectively. We used the NIS-Elements AR 4.3 with N-STORM 3.4 software for acquisition and analysis. Imaging medium was mixed from 80 μl DPBS (Dulbecco’s phosphate-buffered saline), 10 μl MEA (mercaptoethylamine) solution, 10 μl 50% glucose solution and 1 μl GLOX (glucose oxidase) solution. Coverslips with the dried sections were mounted onto microscope slides with 25 μl of freshly prepared imaging medium, and sealed with nail-polish. After selecting the region of interest, confocal image stacks were acquired containing 15 focal planes with 80 × 80 × 150 nm voxel size in *x*, *y* and *z*, respectively. This was followed by bleaching the fluorophores in the STORM channels (561 and 647) with high intensity laser illumination, and running dSTORM acquisition using oblique (near-TIRF) illumination. The acquisition in the two channels was done in sequential mode. Confocal stacks were deconvolved (Huygens Professional), and – together with the STORM molecule lists – processed for further analysis in VividSTORM software (see “Analysis”). Imaging was performed with identical parameters (depth in the section, laser intensities, etc.) for all samples.

### Electron tomography

For the electron tomographic investigation we used 150 nm-thick sections from the hippocampal CA1 region from the anti-vAChT immunogold stained material (see: “Pre-embedding immunoelectron-microscopy”). Before electron tomography, serial sections on single-slot copper grids were photographed with a Hitachi H-7100 electron microscope and a Veleta CCD camera. Serial sections were examined at lower magnification, and vAChT-positive synaptic terminals from the CA1 area selected. For each terminal, the section containing the largest synaptic cross section was chosen for electron tomography. After this, grids were put on drops of 10% HSA in TBS for 10 min, dipped in distilled water (DW), put on drops of 10 nm gold-conjugated Protein-A in DW (1:3) and washed in DW. Finally, we deposited 5 and 5 nm-thick layers of carbon on both sides of the grids. Electron tomography was performed using a Tecnai T12 BioTwin electron microscope equipped with a computer-controlled precision stage (CompuStage, FEI). Acquisition was controlled via the Xplore3D software (FEI). Regions of interest were pre-illuminated for 4–6 min to prevent further shrinkage. Dual-axis tilt series were collected at 2 degree increment steps between −65 and +65 degrees at 120 kV acceleration voltage and ×23,000 magnification with −1.6 to −2 µm objective lens defocus. Reconstruction was performed using the IMOD software package^80^. Isotropic voxel size was 0.49 nm in the reconstructed volumes. After combining the reconstructed tomograms from the two axes, the nonlinear anisotropic diffusion (NAD) filtering algorithm was applied to the volumes. Segmentation of the terminals has been performed on the virtual sections using the 3Dmod software, and measurements were done on the scaled 3D models.

### Isolation of synaptic vesicles

Synaptic vesicles were isolated according to ref. ^81^, with some modifications (Supplementary Figure 4A). Briefly, cortices and hippocampi (neocortex and hippocampus, in 4:1 ratio) of ten C57Bl6 mice (80 days old, mixed gender) were pulverised mechanically after freezing in liquid nitrogen. The resulting fine powder was then homogenised using a Teflon-glass homogeniser in 0.3 M sucrose-containing HEPES buffer (0.3 M sucrose, 50 mM HEPES, pH 7.4, 2 mM EGTA). The homogenate was centrifuged at 100,000×*g* (1 h, 4 °C). The supernatant was laid onto a 0.6 M/1.5 M sucrose step gradient and centrifuged at 260,000×*g* (2 h, 4 °C). Synaptic vesicles were collected from the 0.6 M/1.5 M sucrose solution interface. The vesicles were either immediately used or were frozen in liquid nitrogen and stored at −20 °C for later use.

### Immunostaining and flow cytometry analysis

The vesicles were dialysed overnight, at 4 °C, in phosphate-buffered saline (PBS; 800 ml). For dialysis a 300kD MWCO membrane was used (Biotech CE Dialysis Tubing). All solutions used during the dialysis or subsequent immunostaining were thoroughly filtered through either 0.2 µm (Millipore) or 0.02 µm pore diameter filters (Anodisc-47; GE Healthcare/Whatman). The vesicles were incubated with primary antibody in the presence of 0.1% BSA (RT, 1 h) followed by incubation with secondary antibody (RT, 1 h), under constant shaking. Prior to FACS analysis the stained vesicle suspensions were dialysed again against PBS (800 ml, at RT, for 1 h). FACS analysis was performed on a BD FACSVerse instrument.

### Electron and confocal microscopy of isolated synaptic vesicles

Isolated synaptic vesicle suspension was diluted 10× in TBS, and 100 µl was dropped onto ultra-clean coverslips. After 10 min, 100 µl 4% PFA in 0.1 M PB was added to the drops. For a further 15 min the coverslips were washed gently in distilled water (DW), and processed for TEM and CLSM analysis. For the electron microscopic investigation, vesicles fixed to the coverslips were treated with 1% OsO_4_ in 0.1 M PB at room temperature, dehydrated in ascending alcohol series and in acetonitrile, and embedded in Durcupan (ACM; Fluka). During dehydration, the coverslips were treated with 1% uranyl acetate in 70% ethanol for 20 min. After resin polymerisation, small pieces were cut and removed from the coverslip surface, glued onto plastic blocks, and 40 nm-thick sections were prepared using an ultramicrotome (Leica EM UC6) and picked upon Formvar-coated single-slot grids. Ultrathin sections were examined in a Hitachi 7100 electron microscope equipped with a Veleta CCD camera (Olympus Soft Imaging Solutions, Germany). For CLSM analysis coverslips were washed in PB and Tris-buffered saline (TBS). This was followed by blocking for 1 h in 1% human serum albumin (HSA) dissolved in TBS. After this, sections were incubated in mixtures of primary antibodies for 1 h at room temperature. After incubation, sections were washed in TBS, and were incubated for 1 h at room temperature in the mixture of secondary antibodies, all diluted in TBS. Secondary antibody incubation was followed by washes in TBS, PB, DW and the coverslips were mounted on glass slides with Aqua-Poly/Mount (Polysciences). Immunofluorescence was analysed using a Nikon Eclipse Ti-E inverted microscope (Nikon Instruments Europe B.V., Amsterdam, The Netherlands), with a CFI Plan Apochromat VC 60XH oil immersion objective (numerical aperture: 1.4) and an A1R laser confocal system. The possible influence of chromatic aberration on PSF-position was controlled using fluorescent TetraSpeck™ Microspheres (0.1 µm diameter, blue/green/orange/dark red, ThermoFisher Scientific). We used 405, 488, 561 and 647 nm lasers (CVI Melles Griot), and scanning was done in line serial mode. Image stacks were obtained with NIS-Elements AR software, and deconvolved using Huygens Professional software (www.svi.nl).

### Statistical analysis

During segmentation of tomographic volumes, *z*-scaling was calculated from the thickness difference of the reconstructed volume and the original section thickness, and applied to the 3D models. Mesh surface areas and volumes inside meshed objects were measured with the “imodinfo” programme.

In STORM imaging “molecule lists” were exported from NIS in txt format, and the three image planes of the ics-ids file pairs from the deconvolved confocal stacks matching the STORM-volume were converted to the ome-tiff format using Fiji software. Confocal and corresponding STORM images were fitted in VividSTORM^82^.

When data populations in this work had a Gaussian distribution according to the Shapiro–Wilks *W* test, we reported parametric statistical features (mean ± SD), otherwise we reported non-parametric statistical features (median, interquartile range). For the presentation of electrophysiological data, we used median and interquartile range, because the data did not show Gaussian distribution. Two non-parametric groups were compared using the Mann–Whitney *U* test. Wilcoxon signed-rank test was used for calculating significance between dependent non-parametric data groups. *F*-test was used to compare the variability of data groups. The types of statistical tests used in different investigations are indicated in the text. We always used two-tailed statistical tests. All statistical analyses were carried out using the software package Statistica (StatSoft, Tulsa, OK, USA) or OriginPro 9.2.214 (OriginLab Corporation, Northampton, MA, USA). Adequate sample sizes were chosen based on experience with similar measurements to ensure adequate power to detect a specified effect size. The number of animals used was minimised as much as possible. No animals or measurements were excluded from the measurements after data were collected. Samples from tissue slices or from anatomical sections were collected randomly. The investigator was not blinded to any group allocations, because we investigated only a healthy group of mice. The differences were considered significant at *p* < 0.05.

### Code availability

Custom written codes for sharp wave-ripple and epileptic event detection, and electron tomographic analysis are available upon request.

### Data availability

The data generated during the current study are available from the corresponding author on reasonable request.

## Electronic supplementary material

Supplementary Information
