# Supplementary material for: Co-transmission of acetylcholine and GABA regulates hippocampal states
Source: Nat Commun. 2018 Jul 20;9:2848. doi: 10.1038/s41467-018-05136-1 (PMC6054650; doi:10.1038/s41467-018-05136-1)
Supplement: Supplementary file 1 — Supplementary Information [file 41467_2018_5136_MOESM1_ESM.pdf]

SUPPLEMENTARY INFORMATION FOR

Co-transmission of acetylcholine and GABA regulates hippocampal states

Virág T. Takács, Csaba Cserép, Dániel Schlingloff, Balázs Pósfai, András Szőnyi, Katalin E. Sos, Zsuzsanna Környei, Ádám Dénes, Attila I. Gulyás, Tamás F. Freund, Gábor Nyiri

Correspondence to: Gabor Nyiri, nyiri@koki.hu

This PDF file includes:

- Supplementary Note 1-8:
- Supplementary Discussion
- Supplementary Figures 1-4
- Supplementary Tables 1-4
- Supplementary References

Supplementary Note 1:

Cortical S1 cholinergic axon terminals also form synapses

In somatosensory cortex S1, similar to hippocampal cholinergic fibres all cholinergic terminals established synapses (Supplementary Figure 1A, axon J-L). In somatosensory cortex S1, differentiation of pyramidal and interneuronal dendritic shafts is not possible based on electron microscopic profiles. In S1 cortex, cholinergic terminals targeted dendrites (54%), spines (38%), while 8% of their synaptic targets remained unidentified (Supplementary Table 4).

Supplementary Note 2:

Synapses established by cholinergic fibres express $GABA_A$ receptors and its scaffolding protein postsynaptically

Previously, we demonstrated that synapses established by cholinergic fibres (in CA1, S1 somatosensory cortex, prefrontal cortex, basolateral amygdala and centrolateral thalamic nucleus) expressed the postsynaptic protein NL2 [1]. This protein directly interacts with gephyrin, a core scaffolding protein of inhibitory postsynaptic densities [2] and their complex is implicated in the anchoring and clustering of $GABA_A$ receptors postsynaptically [3–5]. Here, we labelled cholinergic fibres either with vesicular acetylcholine transporter (vAChT, in WT mice) or eYFP (in ChAT-Cre mice, where medial septum was injected with a Cre-dependent eYFP-expressing AAV). Latter labelings were developed with DAB, while gephyrin or $GABA_A$ receptor γ2 subunits were labelled with immunogold particles. Using electron microscopy, we tested fully reconstructed synapses of vAChT or eYFP-AAV labelled terminals for the presence of immunogold particles. Data from vAChT and AAV-eYFP labelled samples were not statistically different; therefore, they were pooled.

We found that at least 81% of 188 synapses collected in the CA1 area of two WT and two ChAT-Cre mice contained gephyrin postsynaptically (Supplementary Table 3). Immunogold particles in these synapses were associated with the cytoplasmic side of the postsynaptic membrane of the innervated pyramidal cell dendrites (Figure 1B-D, G-I) and spines (Supplementary Figure 1B) or interneuron dendrites (Figure 1J). The antibody used for $GABA_A$ receptor-labelling was directed against an extracellular epitope of the γ2 subunit [6,7] and labelled synaptic clefts accordingly. In the CA1 of three WT and two ChAT-Cre mice, at least 80% of the synapses established by cholinergic fibres (out of 172) showed $GABA_A$ receptor γ2 subunit labelling (Supplementary Table 3). Cholinergic fibres established $GABA_A$ receptor containing synapses on both dendrites (Figure 1E,F,K,L,N; Supplementary Figure 1 C, D) and spine-necks (Figure 1M). In addition, we found that at least 83% of synapses of vAChT-positive terminals in the somatosensory cortex S1 (n=36, Supplementary Figure 1 E-F) contained these $GABA_A$ receptor γ2 subunits. Because reliable nicotinic receptor antibodies are not available, they could not be localized directly.

Supplementary Note 3:

Cholinergic terminals possess the molecular machinery for GABA-release

By crossing a zsGreen fluorescent reporter-mouse-line with a vesicular GABA transporter (vGAT)-Cre mouse line, we created mice, in which all GABAergic cells are zsGreen labelled (vGAT-zsGreen mice). After co-labelling medial septum sections for ChAT, we found that all cholinergic cells were also positive for zsGreen (n=243 cells in 2 mice, Figure 2A, B), while many of the cells were positive only for zsGreen, corresponding to the non-cholinergic, GABAergic neurons of the

medial septum. These results confirmed that all hippocampal projecting cholinergic cells express vGAT.

To confirm that septo-hippocampal cholinergic fibres can synthetize GABA, we performed immunofluorescent reactions against glutamate-decarboxylase (GAD65) and eYFP on hippocampal sections of ChAT-Cre mice, in which medial septal cholinergic fibres were labelled with Cre-dependent eYFP-AAV. We confirmed the GAD65 expression in eYFP positive terminals (Figure 2C) as well.

To confirm vGAT protein expression in cholinergic terminals we performed vGAT-eYFP and vAChT-eYFP multiple labelings, and found that vGAT was present in the majority of the eYFP positive cholinergic terminals (at least 82.9%, n=311 in 3 mice Figure 2D). We also quantified vAChT expression, which was found to be present in at least 63.6% of the eYFP positive cholinergic terminals (n=364 in 3 mice).

Using postembedding GABA-immunogold staining, we also tested, whether cholinergic terminals contain GABA itself (Figure 2 E-F). We measured postembedding GABA-immunogold labelling densities in preembedding labelled vAChT-positive terminals (n=2 mice, 24 terminals). Background level was estimated measuring gold particles on vAChT-negative, putative glutamatergic terminals that formed asymmetric synapses in the vicinity of the examined vAChT-positive terminals (n=2 mice, 34 terminals). Data from two mice were not different statistically; therefore, they were pooled. We found a significantly higher level of immunogold labelling for GABA in vAChT-positive terminals than in glutamatergic terminals (3.3 times higher density; Figure 2F), suggesting the presence of GABA in these terminals.

Supplementary Note 4:

Synaptic vesicles of cholinergic terminals are highly heterogeneous and relatively large

We analysed the volume and elongation of vesicles [8,9] in cholinergic terminals along with similar data from purely GABAergic terminals (Figure 4E). As expected, GABAergic vesicles were small and elongated (median volume: 13730 nm^3, 11174-16434 nm^3 interquartile range; median of elongation factor: 2.90, 2.52-3.41 interquartile range; n=54 vesicles from 2 mice). However, the volume of cholinergic vesicles were significantly larger (Mann-Whitney test, p<0.001). The volume of the vesicles in cholinergic terminals showed a significantly higher variability as well (F-test, p<0.001), ranging from the very large and round ones to the small and elongated vesicles (median volume: 23267 nm^3, 19160-27539 nm^3 interquartile range; median elongation factor: 1.80, 1.57-2.04 interquartile range; n=140 vesicles from two mice, Figure 4E). The small and elongated vesicles in the cholinergic terminals were similar to the purely GABAergic ones from GABAergic interneuron terminals (Figure 4E). These data suggest that cholinergic terminals contain both smaller, more elongated GABAergic vesicles and larger, rounder cholinergic vesicles, both of which are released from the same synaptic active zone. Interestingly, some vesicles that were directly labelled with vAChT-immunogold particles were rather large and round (Figure 4E). We also observed a vAChT-labelled vesicle, fused to the synaptic membrane, likely releasing its transmitter into the synaptic cleft (Figure 4C). In subsequent experiments, we collected further evidence that acetylcholine and GABA are filled into different vesicles allowing their separately-regulated co-transmission.

Supplementary Note 5:
Acetylcholine and GABA are released at the same active zone in cholinergic terminals

We also tested, whether the two transmitter systems use the same or distinct active zones. We performed multiple immunofluorescent labelling experiments on virus labelled (eYFP) cholinergic fibres in ChAT-Cre mice for gephyrin, vAChT and eYFP, followed by confocal fluorescent imaging. We observed gephyrin-labelled puncta opposed to eYFP-positive terminals (Figure 4G, H), identifying the postsynaptic active zones of these fibres. vAChT-labelling was clearly concentrated opposite to the gephyrin-puncta, proving a tight association to the synaptic active zones (Figure 4G, H). Scale-free analysis confirmed that the likelihood of vAChT labelling was the highest at the synaptic active zones (Figure 4I; n=32 synapses from two mice). To directly examine the existence of a mixed cholinergic/GABAergic vesicle pool, we labelled brain slices for vGAT, vAChT and eYFP, and performed correlated fluorescent confocal laser scanning microscopy (CLSM) and superresolution STORM imaging (Figure 4J). The superresolution images confirmed that vAChT- and vGAT-labelled vesicle pools overlap, and were localized to the same small, confined portions of the eYFP septo-hippocampal terminals.

Supplementary Note 6:
Acetylcholine and GABA are released from different vesicles in cholinergic terminals

Although a previous study in rat has suggested that GABA-containing synaptic vesicles do not contain acetylcholine [10], using a highly specific method, we confirmed that GABA and acetylcholine vesicular transporters are localized on different vesicles in mouse cortical axon terminals. We used isolated synaptic vesicles to test whether acetylcholine and GABA are packed into the same vesicles. Isolation from neocortex and hippocampus was performed according to Mutch et al. (Supplementary Figure 4A,[11]). Isolated vesicles were investigated by flow cytometry for synaptophysin (SYP) expression. Labelling with a specific SYP antibody resulted in an about two orders of magnitude higher mean fluorescent intensity of vesicle preparations compared to the labelling with the secondary antibody alone (Supplementary Figure 4B), suggesting a highly purified preparation. After fixation and dehydration of vesicle preparations, we confirmed the presence of synaptic vesicles surrounded by lipid bilayer on electron microscopic images (Supplementary Figure 4E). The analysis confirmed that the diameter of the isolated vesicles was 37.55 nm (median, 33.78-40.21 nm interquartile range, n=100 vesicles; Supplementary Figure 4C), in accordance with literature data [12]. Next, we performed immunolabelling experiments on isolated synaptic vesicles fixed onto coverslips. Prior to CLSM imaging, we labelled the samples for SYP, vGAT, vAChT and vesicular glutamate transporter (VG1). As expected, we observed well separated fluorescent dots (point-spread functions, PSF) of the fluorophores in one single focal plane (Supplementary Figure 4F), but most PSFs showed vesicular co-localization of one of the vesicular transporters and SYP. Control experiments of the immunolabelling confirmed the lack of unspecific staining (Supplementary Figure 4I). In the absence of vesicle suspension, no PSFs were found in the CLSM scans, and the exclusion of any primary antibody led to the selective disappearance of PSFs in the corresponding channel. We also tested the distribution of fluorescent PSFs on the CLSM images. SYP-labelled vesicles were usually more than 1 µm away from each other as nearest-neighbor analysis of PSF centroids confirmed (median: 1.16 µm, 0.82-1.59 µm interquartile range, 0.46-3.92 min-max, n=149 vesicles; Supplementary Figure 4D). When PSFs in different channels colocalized, their centroids were never farther away from each other than 0.130 µm (median: 0.03 µm, 10-50 µm interquartile range, 0-0.13 min-max, n=92 vesicles; Supplementary Figure 4D). These experiments confirmed that co-localizing PSFs correspond to a

single vesicle. Next, we analysed co-localizations of the PSFs in different channels (Supplementary Figure 4G) and found that 29.2% of vesicles were labelled only for SYP, 44.5% were double-labelled for VG1 and SYP, 14.3% were double-labelled for vGAT and SYP, 11.1% were double-labelled for vAChT and SYP. Only a negligible amount of vesicles (0.9%) were triple labelled with any combinations, whereas only a sub-fraction of these vesicles (0.14% of all) were co-labelled for vAChT and vGAT. Only 0.98% of all vGAT/SYP positive vesicles were labelled for vAChT, and only 1.26% of all vAChT/SYP positive vesicles were labelled for vGAT. These numbers are in the range of false positive labelling as confirmed in the control experiments, where primary antibodies were omitted (Supplementary Figure 4I). These data suggest that vesicular transporters for glutamate, GABA and acetylcholine are expressed by distinct vesicle populations in cortical samples (Supplementary Figure 4H; n=353 vesicles). Therefore, acetylcholine and GABA may be released at the same active zones, but from different vesicles.

Supplementary Note 7:

Identification of basal forebrain cholinergic fibres in the hippocampus: control experiments

We either used immunolabelling against the vesicular acetylcholine transporter (vAChT), or performed anti-eYFP staining on sections from ChAT-Cre mice, the medial septal areas of which have previously been injected with Cre-dependent eYFP-adeno associated virus (AAV). Both of these methods had to be verified for selectivity and specificity, thus we completed a comprehensive set of control experiments. The cholinergic innervation of the hippocampus is reported to originate exclusively from the basal forebrain. Although the presence of a local cholinergic cell population in the mouse hippocampus was reported to be an artefact [13] we also tested for it. We injected Cre-dependent eYFP-AAV into the hippocampi of ChAT-Cre mice (Supplementary Figure 2C, inset), and stained hippocampal sections for eYFP, vAChT and vGAT (Supplementary Figure 2E, F). We found a few eYFP positive cells in the hippocampus. They were extremely rare and resembled dentate gyrus granule cells and CA3 pyramidal cells. We also found some sparsely distributed eYFP positive fibres originating from them, but vAChT or vGAT immunoreactivity was never found in these eYFP positive terminals (0 out of 323 terminals, from 2 mice, Supplementary Figure 2F). We also tested vAChT positive terminals in the same samples, and never found any eYFP-positivity in them (0 out of 3673 from 2 mice, Supplementary Figure 2F). Thus, we confirmed that there are no cholinergic cells in the hippocampus, only some extremely rare ectopic expression of the Cre enzyme. These results also confirmed that we can reliably label the septo-hippocampal cholinergic fibres with vAChT labelling.

To verify the other approach, we injected eYFP-AAV into the medial septal areas of ChAT-Cre mice (Supplementary Figure 2C), and performed PV/ChAT/eYFP triple labelings (Supplementary Figure 2D). 97.6% of all tested eYFP positive cells in the MS were also positive for ChAT (the few % of false negative cells are likely due to not perfectly efficient antibody penetration), but none of them were positive for PV (n=212 in 2 mice). We also tested the fibres of these cells in the hippocampus, and performed a PV/vAChT/eYFP triple labelling (Supplementary Figure 2A, B). We found that eYFP positive terminals colocalized with vAChT-labelling, but were never positive for PV (n=252 terminals from 2 mice). These results confirmed that eYFP positive fibres in these animals originate exclusively from cholinergic cells.

Supplementary Note 8:

Statistical details for Figures

Figure 2F: Medians (columns) and interquartile ranges (bars) of immunogold densities of GABA labelling in glutamatergic (Glut, median: 3.5 gold particles/ µm2, interquartile ranges: 1.5-5.3) and in VAChT-positive terminals (VAChT, median: 11.5 gold particles/ µm2, interquartile ranges: 6.8-22.7). Asterisk indicates significant difference (Mann-Whitney Test: $p < 0.05$). vAChT-negative terminals forming type I synapses were considered to be glutamatergic.

Figure 3H: Amplitude, 20-80% rise time and decay time of unitary GABAergic IPSCs from pyramidal cells (n=5) and inhibitory neurons (n=16). Box plots represent median values, with interquartile ranges, whiskers represent min/max values. Amplitude in pA: PCs: 37.28 (20.94, 61.61); INs: 61.56(46.78, 98.39), Mann-Whitney Test: $p<0.05$. Rise time (in ms) in PCs: 2.06 (1.62, 2.29), INs: 1.29 (1.12, 1.83); Mann-Whitney Test: not significant. Decay time (in ms): PCs: 16.29 (15.72, 25.94), INs: 11.35 (8.68, 14.10), Mann-Whitney Test: $p<0.05$.

Figure 3I: Averages of IPSC amplitudes for the 5 pulses presented on panel G show strong short-term depression (STD) of GABAergic transmission evoked by stimulating cholinergic fibers. **PCs:** 2 Hz: 1st –42.89 (±21.79); 2nd –25.74 (±13.7); 3rd –25.63 (±14.85); 4th –24.65 (±18.64); 5th – 21.54 (±17.04). 5 Hz: 1st – 40.12(±18.32); 2nd – 22.96(±12.17); 3rd –18.68 (±10.9); 4th – 20.45 (±14.9); 5th – 19.36 (±17.28). 10 Hz: 1st – 38.59(±13.62); 2nd – 19.04(±12.67); 3rd – 13.65(±8.57); 4th – 12.44(±7.5); 5th – 11.46(±7.44). 20 Hz: 1st –37.10 (±24.18); 2nd –11.98 (±12.24); 3rd –9.82 (±10.35); 4th – 9.13(±8.92); 5th – 6.78 (±8.06). **INs**: 2 Hz: 1st – 66.72(±18.33); 2nd – 51.53(±19.44); 3rd – 43.19(±15.35); 4th – 41.44(±13.34); 5th – 36.78(±13.36). 5 Hz: 1st – 70.55(±18.31); 2nd – 51.73(±16.27); 3rd – 39.02(±15.12); 4th – 32.58(±15.49); 5th – 32.76(±13.22). 10 Hz: 1st – 70.01(±14.75); 2nd – 50.89(±14.89); 3rd – 32.75(±14.93); 4th – 29.50(±13.82); 5th – 30.02(±14.41). 20 Hz: 1st – 67.1(±15.22); 2nd –35.0 (±15.22); 3rd –28.76 (±15.07); 4th – 23.68(±14.38); 5th – 21.55(±13.14).

Figure 5B: IPSP amplitude at control (median (interquartile range)): 0.88 mV (0.78-1.29), atropine: 1.35 mV (1.13-1.38); Wilcoxon-sign rank test: $p<0.05$. EPSP amplitude at control: 0.42 mV (0.27-0.77), atropine: 0.65 mV (0.45-2.22); Wilcoxon-sign rank test: $p<0.05$.

Figure 5C: IPSP amplitude at control: 0.74 mV (0.43-1.05), AFDX-116: 1.08 mV (0.58-1.42); Wilcoxon-sign rank test: $p<0.05$; EPSP amplitude at control: 0.17 mV (0.1-0.27), AFDX-116: 0.29 mV (0.18-0.74); Wilcoxon-sign rank test: $p<0.05$.

Figure 5D: IPSP amplitude at control: 0.79 mV (0.57-1.05), CGP: 0.9 mV (0.79-1.24); Wilcoxon sign rank test: $p<0.05$; EPSP amplitude at control: 0.24 mV (0.13-0.25), CGP: 0.37 mV (0.23-0.46); Wilcoxon sign rank test: $p<0.05$.

Figure 5E: IPSP amplitude at control: 1.21 mV (1.01-1.56), ω-agatoxin: 0.85 mV (0.59-0.96); Wilcoxon sign rank test: $p<0.05$. EPSP integral at control: 0.29 mV*s (0.24-0.94); agatoxin: 0.34 mV*s (0.21-0.64), Wilcoxon sign rank test: $p=0.63$.

Figure 5F: EPSP integral at control: 0.55 mV*s (0.25-0.59); conotoxin: 0.13 mV*s (0.11-0.25); Wilcoxon sign rank test: $p<0.05$. IPSP amplitude at control: 0.74 mV (0.49-0.91), conotoxin: 0.68 mV (0.51-0.72); Wilcoxon sign rank test: $p=0.52$.

Figure 6D: SWR rate in the absence of cholinergic blockers: 1.65 Hz (1.18-1.78) at control, 1.18 Hz (0.99-1.27) during illumination and 1.78 Hz (1.27-1.95) during recovery period, Wilcoxon-sign rank test: control-stimulation, $p<0.05$; stimulation-recovery, $p<0.01$; control-recovery, $p<0.05$.

Figure 6F: SWR rate in cholinergic blockers: 1.0 Hz (0.93-1.34) at control, 0.44 Hz (0.40-0.99) during illumination, 0.95 Hz (0.79-1.33) during recovery period, Wilcoxon-sign rank test: control-stimulation, p<0.01; stimulation-recovery, p<0.01; control-recovery, not significant.

Figure 6I: Epileptic discharge rate in the absence of AChR blockers: 0.61 Hz (0.47-0.68) at control, 0.54 Hz (0.44-0.58) during illumination, 0.62 Hz (0.5-0.63) during recovery period, Wilcoxon-sign rank test: control-stimulation, p<0.05; stimulation-recovery, p<0.05; control-recovery, not significant.

Figure 6K: Epileptic discharge rate in the presence of AChR blockers: 0.56 Hz (0.49-0.60) at control, 0.43 Hz (0.37-0.44) during illumination, 0.48 Hz (0.42-0.54) during recovery period, Wilcoxon-sign rank test: control-stimulation, p<0.01; stimulation-recovery, p<0.05; control-recovery, not significant.

Supplementary Discussion:

Cholinergic non-synaptic neurotransmission.

For decades, the predominant form of cholinergic communication was thought to be a form of “non-synaptic volume transmission" [14–20], which was supported by electron microscopic studies showing that cholinergic terminals form few synapses [3-17%: in cat striate cortex [21], rat parietal cortex [22,23], rat hippocampus [24,25], mouse hippocampus [26]], while some papers reported more frequent synapses [44-67%: in macaque prefrontal cortex [27], human temporal lobe [28], rat parietal cortex [29]]. Although acetylcholine esterase (AChE) was known to be highly effective in terminating extracellular cholinergic signal, the presence of certain extrasynaptic acetylcholine receptors (”receptor mismatch“,[26,30]) suggested that extracellular diffusion of acetylcholine occurs. Micro-dialysis experiments [31] and the localization of AChE, distant from cholinergic terminals, also seemed to support a non-synaptic “volume” transmission hypothesis [14]. However, later, highly sensitive microelectrodes showed faster, phasic changes in extracellular acetylcholine levels that facilitated cue detection and cortical information processing [20,32–35]. Basal forebrain cholinergic neurons were also shown to respond to reward and punishment with extremely high speed and precision [36], and recent data suggested that cholinergic cells may regulate cortical information processing with a remarkable, millisecond-scale temporal precision [34,37–39]. However, such a delicate temporal precision is hard to imagine without synapses and it remained inconclusive, whether the mode of acetylcholine signalling is synaptic “wired” transmission or “non-synaptic volume” transmission by ambient acetylcholine [26].

GABAergic markers in cholinergic cells

Basal forebrain cholinergic cells share a common developmental origin with different populations of cortical, striatal and basal forebrain GABAergic neurons [40–43]. Previous studies have suggested that less than 2% of cholinergic cells express GABAergic markers [44,45], while about 8% of ChAT-positive boutons in the cat striate cortex was shown to contain GABA [46]. The recognition of GABAergic signalling in the BF cholinergic system may have been hampered by its lack of GAD67 [47] and GABA transporter 1 [48]. Although the associations of cholinergic terminals with gephyrin [49] and NL2 [1] suggested the capability of GABAergic signalling from these terminals. While, for example, GABA is released together with glutamate or aspartate in the hippocampus, or with dopamine in periglomerular cells [50,51], and acetylcholine is released with glutamate in striatum [52]; GABA and acetylcholine were also shown to be released together in retina and frontal cortex [45,47,53,54]. However, the precise architecture, the mechanism of the dual cholinergic/GABAergic transmission and their hippocampal synaptic physiological and network effects have not yet been investigated.

SUPPLEMENTARY FIGURES 1-4:

Supplementary Figure 1,

Cholinergic axons establish synaptic contacts just as frequently as other GABAergic fibers and express GABAergic postsynaptic markers

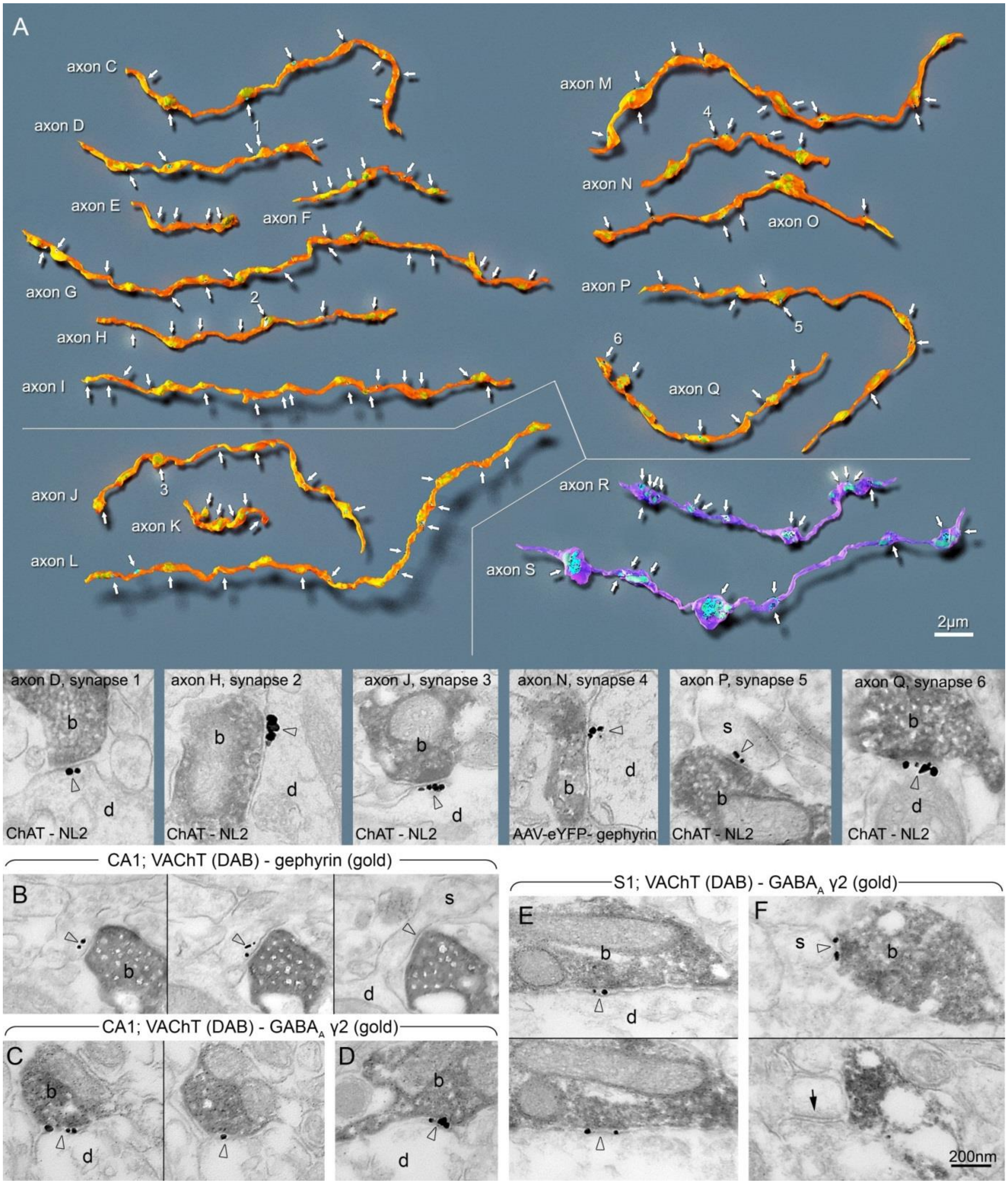

A: 3D EM reconstructions of DAB-labelled ChAT-positive (axons C-L; P,Q), AAV-eYFP virus-traced septo-hippocampal (axons M-O) and CB_1-positive (axons R, S) axonal segments from the hippocampus (str. ori: axons C-F, M-O, R; str. rad: G-I, S; str. l-m: P,Q) and layer I-III of the somatosensory cortex (axons J-L). Gold

labelings of NL2 (axons C-L; P-S) or gephyrin (axons M-O) were used to recognize synapses (arrows). Notice, that the linear density of synapses along cholinergic (C-Q) and CB_1-positive GABAergic axons (R,S) are not different (see also data in Figure 1). Electron micrographs show cholinergic terminal boutons (b) forming synapses 1-6 (arrowheads, indicated by the same numbers in the 3D-reconstructions) on dendrites (d) and a spine (s).

B-F: Electron micrographs from combined preembedding immunogold/immunoperoxidase experiments for gephyrin or $GABA_A\gamma 2$ receptor subunit (immunogold) and vAChT (DAB: dark, homogenous reaction product) reveal the presence of gephyrin postsynaptically (B, arrowheads) and $GABA_A\gamma 2$ receptor subunit (C, D; arrowheads) in the synaptic cleft of synapses established by vAChT-positive axons in the hippocampus (CA1, str. l-m: B, str. ori: C, D). E-F: vAChT-positive terminals (DAB) establish synapses with $GABA_A\gamma 2$ receptor subunits (immunogold, arrowheads) in the neocortex S1 area (E-F). Two or three consecutive sections of the same synapses are shown in B, C, E, and F. Labelled terminals shown in the EM images innervate dendrites (d) or spines (s). Spine in F receives a type I synapse (arrow in the lower panel) from an unlabelled terminal. Scale bars are 2 µm for all reconstructions and 200 nm for all EM images.

Supplementary Figure 2,

Control experiments for the labelling of septo-hippocampal cholinergic cells and fibres with Cre-dependent viral and immunolabelling techniques

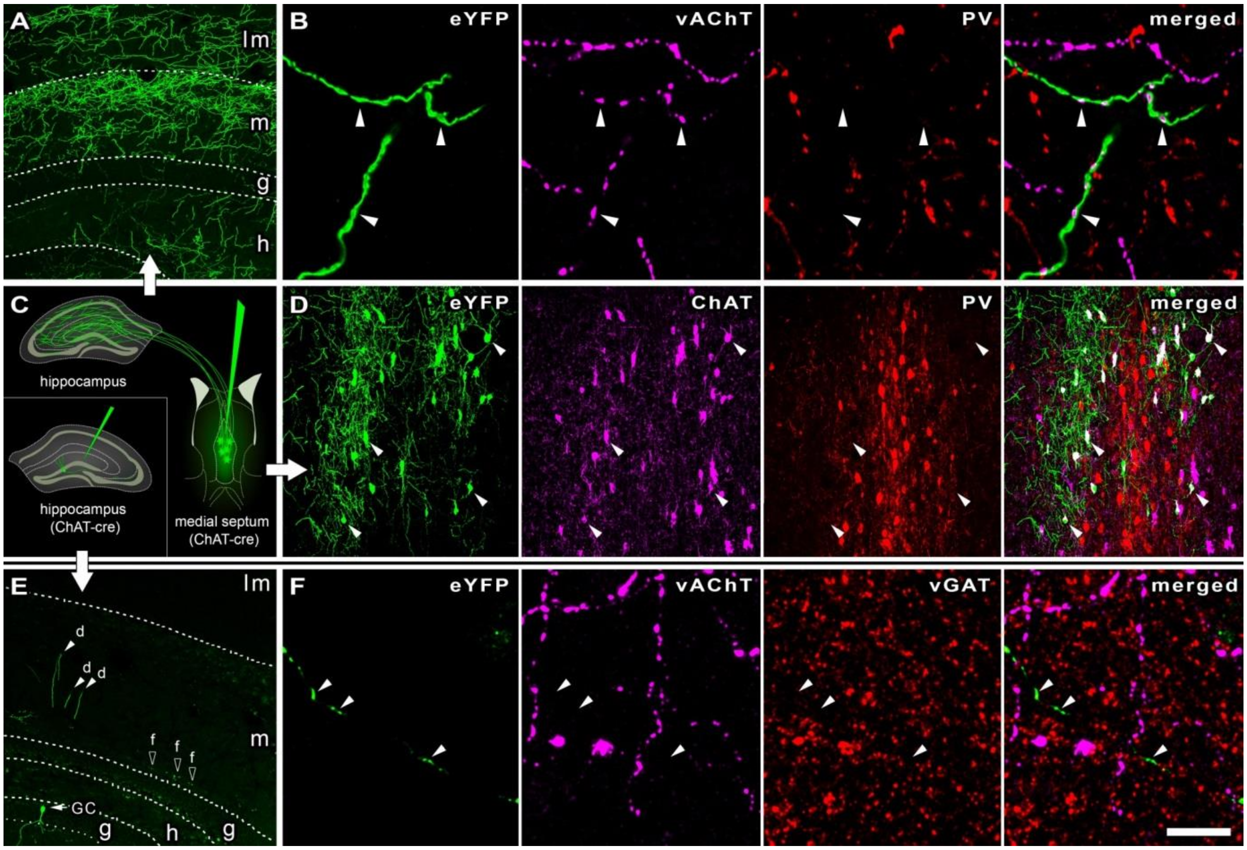

A: A robust network of eYFP-expressing cholinergic fibres is present in the hippocampus after AAV-injection into the MS of ChAT-Cre mouse. (lm: laconosum-moleculare, m: moleculare, g: granule-cell layer, h: hilus)

B: Confocal laser scanning microscopy images confirm that AAV-eYFP virus-traced septo-hippocampal fibres contain vAChT, but not parvalbumin (PV) in the hippocampus, demonstrating that septal GABAergic PV cells did not express Cre-dependent fluorescent protein. vAChT-labelling is localized to the terminals of the fibres. (Arrowheads mark the position of some terminals.)

C: Schematic diagram showing the AAV-eYFP-injections into the MS or hippocampus (inset) of ChAT-Cre mice.

D: All eYFP-expressing MS neurons were positive for ChAT, while none of them contained PV. (Arrowheads mark the position of some cell bodies.)

E: After AAV-eYFP-injection into the hippocampus of ChAT-Cre mice, a negligible amount of cells could be detected to express eYFP. eYFP-expressing granule cell can be seen with some dendrite-segments and few scattered fibres. (d: dendrite, f: fibre, GC: granule-cell)

F: Local eYFP-expressing fibres in the hippocampus do not contain vAChT or vGAT (Arrowheads mark some terminals, scale bar on F is 150 µm for A, D, E, and 6 µm for B and F), demonstrating the lack of cholinergic fibres originating from inside the hippocampus.

Supplementary Figure 3,
GABAergic short-term depression is a presynaptic property of cholinergic fibers.

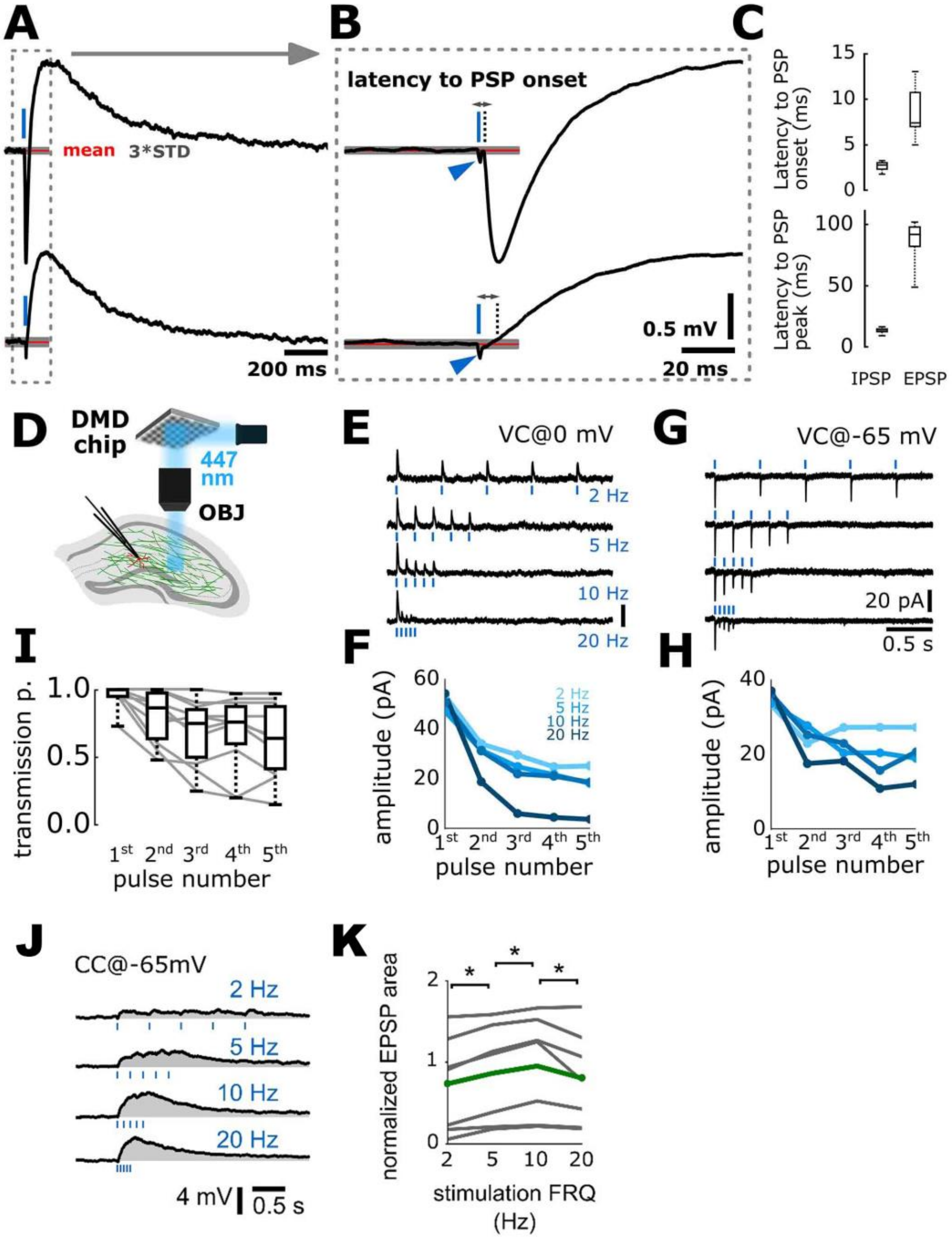

A: Average membrane potential response of an inhibitory neuron recorded in str. lacunosum-moleculare for cholinergic fiber stimulation (top). Latency from stimulation start to PSP onset was calculated as the time when the signal crosses 3 times standard deviation of baseline (orange line and grey shaded area represent mean and 3 times STD of the baseline). Inhibition of $GABA_A$Rs (10 µM gabazine) blocks hyperpolarization (bottom) and the latency of the cholinergic response can be calculated similarly as well.
B: Responses are magnified in time (from panel A). Blue arrowheads mark photoelectric artefacts evoked by 1 ms optical stimulus (blue bar). Note the short latency to rise (dotted line) in both the GABAergic IPSP and cholinergic EPSP.

C: Latency from stimulus start to PSP onset (top) and PSP peak (bottom) are shown. IPSP time to onset (n = 7, in ms): 2.8 (2.2, 3.1), time to peak: 13.8 (12.7, 14.9). EPSP time to onset (n = 6, in ms): 7.4(7.0, 11.7), time to peak: 92.0 (80.5, 98.0).

D: Channelrhodopsin 2 expression in axon terminals could change the short-term plasticity of the examined synapse by illumination driven calcium entry through the light activated channels. To exclude this possibility, with the help of a digital micro-mirror device (DMD), we have illuminated only axons running towards the measured cell, but not the axon terminals themselves.

E: We have recorded from inhibitory neurons in str. lacunosum moleculare (n=5), and illuminated the slice far (~500 µm) from the cells. Similarly as in Figure 3, we applied 5 pulses at different frequencies (2, 5, 10 and 20 Hz). Averaged traces from one cell are shown.

F: Mean amplitudes for different frequencies are shown from 5 cells. GABAergic currents evoked by light stimulation using the DMD device show similar short-term depression as presented on Figure 3. Amplitudes in pA (mean±std): 2 Hz: 1st – 53.41(±23.66); 2nd – 34.05(±17.79); 3rd – 29.59(±11.54); 4th – 24.80(±9.47); 5th – 25.20(±11.54). 5 Hz: 1st – 47.05(±21.15); 2nd – 31.53(±15.58); 3rd – 24.87(±12.68); 4th – 21.69(±10.39); 5th – 18.20(±9.58). 10 Hz: 1st – 50.66(±19.43); 2nd – 31.22(±13.31); 3rd – 22.00(±8.16); 4th – 21.10(±10.21); 5th – 18.63(±4.71). 20 Hz: 1st – 54.21(±18.28); 2nd – 18.80(±3.95); 3rd – 6.07(±1.8); 4th – 4.55(±1.82); 5th – 3.75(±2.11).

G: Another factor, which can contribute to short-term depression is postsynaptic chloride loading and subsequent reduction of chloride drive, due to series of stimuli. This phenomenon can be explored with reversing chloride gradient, as demonstrated here by using a high chloride content intracellular solution. Average GABAergic responses are shown from one cell.

H: Mean amplitudes for different frequencies are shown from 5 cells. GABAergic responses show similar short-term depression as described previously. Amplitudes in pA (mean±std): 2 Hz: 1st – 33.27(±19.16); 2nd – 23.02(±23.76); 3rd – 27.28(±16.80); 4th – 27.33(±20.33); 5th – 26.31(±25.86). 5 Hz: 1st – 33.27(±14.57); 2nd – 27.76(±17.51); 3rd – 20.46(±9.24); 4th – 22.48(±10.67); 5th – 10.00(±13.91). 10 Hz: 1st – 36.23(±24.69); 2nd – 25.29(±20.18); 3rd – 23.02(±20.10); 4th – 15.79(±10.36); 5th – 20.76(±17.03). 20 Hz: 1st – 37.09(±33.27); 2nd – 17.61(±20.65); 3rd – 18.29(±24.65); 4th – 10.95(±10.16); 5th – 12.10(±11.49).

I: Transmission probability is shown for 10 Hz stimulation from the 10 cells presented on Figure E-H. The decrease in transmission probability support presynaptic mechanism for STD. Transmission probability: 1st pulse 1(0.95, 1), 2nd pulse: 0.87 (0.60, 1), 3rd pulse: 0.75 (0.45, 0.87), 4th pulse: 0.76 (0.55, 0.90), 5th pulse: 0.64 (0.37, 0.90). Wilcoxon-sign rank test: 1st–2nd: $p<0.05$, 2nd–3rd: $p<0.05$, 3rd–4th and 4th-5th: not significant. In some cells, transmission probability remained stable despite the observed STD in IPSC amplitude, suggesting that multiple contacts were excited and "averaged" by optical illumination. These results support our hypothesis that GABAergic short-term depression emerges presynaptically, and not the result of channelrhodopsin-2 expression or postsynaptic chloride loading.

J: Short-term dynamics of cholinergic EPSPs recorded from inhibitory neurons in str. lac.-mol. in response to 5 light pulses at different frequencies (top; 2, 5, 10, 20 Hz, n = 7). Cholinergic events overlap, not allowing reliable EPSP peak detection. Therefore, STP was quantified as the integral of the evoked events.

K: The relative change in EPSP integral for the stimulations at different frequencies (n=7, average is green). Unlike the GABAergic component (Figure 3), short-term depression was not observed in cholinergic responses. Slight increase in EPSP area from 2 to 10 Hz suggests some form of a weak facilitation of the cholinergic component. EPSP integral at 2 Hz: 2.19 mV*s (0.41-3.63), 5 Hz: 2.56 mV*s (0.49-3.70), 10 Hz: 2.92 mV*s (0.53-3.88), 20 Hz: 1.82 mV*s (0.46-3.92); Wilcoxon-sign rank test: $p<0.05$; 0.05; 0.03.

Supplementary Figure 4,
Vesicular acetylcholine and GABA transporters are localized on different vesicles

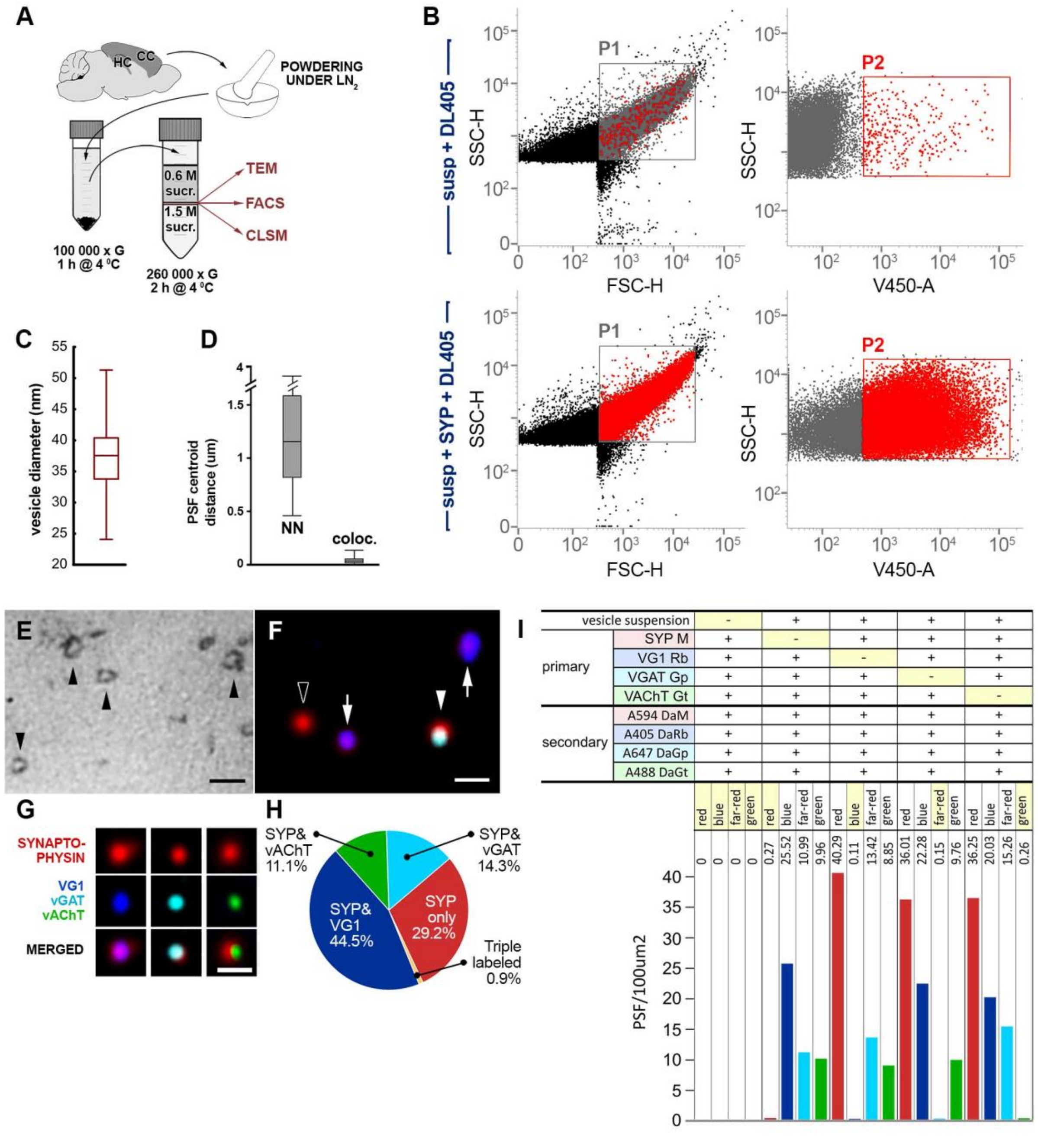

A: Schematic drawing depicts the main steps of synaptic vesicle isolation. Cerebral cortices (CC) and hippocampi (HC) of mice were frozen and powdered under liquid nitrogen. After homogenization, the sample was centrifuged at 100 000 G (1h, 4°C). The supernatant was laid onto a 0,6M/1,5M sucrose step gradient and centrifuged at 260 000 G (2h, 4°C). Synaptic vesicles were collected from the 0,6M/1,5M sucrose solution interface, and processed for transmission electron microscopy (TEM), flow cytometry (FACS) and confocal laser scanning microscopy (CLSM).

B: Flow cytometric analysis of isolated synaptic vesicles from mouse hippocampal and neocortical areas show the purity of our vesicular sample preparations. Dot plot diagrams show putative vesicles

investigated without (upper row) and with (lower row) synaptophysin (SYP) primary antibody labelling, followed by fluorescent secondary antibody labelling in both cases. Dot plots on the right, show strong fluorescence signal of SYP immunolabelled putative vesicles (SSC/V450), gated also on the SSC/FSC dot plots [P1], suggesting that the isolated sample is highly purified.
C: Diameter distribution of isolated synaptic vesicles on the TEM images show typical vesicular diameters.
D: Distribution of fluorescent point-spread functions on the CLSM images. SYP labelled vesicles were usually more than 1 µm away from each other as nearest-neighbor analysis of PSF centroids confirmed. Centroids of PSF in different channels belonging to one vesicle were never farther away from each other than 130 nm.
E: Electron micrograph of fixed and dehydrated synaptic vesicles isolated (see A) from cerebral cortex and hippocampus. Arrows point to individual vesicles, scale bar is 100 nm.
F: CLSM image of fixed and immunolabelled isolated synaptic vesicles. Arrows point to point spread functions (PSF) of VG1 and SYP co-labelled vesicles, arrowhead marks PSF of vGAT and SYP co-labelled vesicle, empty arrowhead shows PSF of vesicle positive only for SYP. Scale bar is 400 nm.
G: CLSM images of individual PSFs representing synaptic vesicles from quadruple labelling experiments. Scale bar is 600 nm.
H: Percentages of differently labelled synaptic vesicles (n=350 individual vesicles). Frequency of triple labelled vesicles were less than 1 percent, confirming that vesicular transporters for glutamate, GABA and acetylcholine are expressed by distinct vesicle populations.
I: Control experiments of the immunolabelling method confirm the lack of non-specific staining. In the absence of vesicle suspension no PSF-s were found in the CLSM scans, and the exclusion of each primary antibody led to the selective disappearance of PSFs in the corresponding channel.

SUPPLEMENTARY TABLES 1-4:

Supplementary Table 1 (primary antibodies)

RAISED AGAINST	RAISED IN	DILUTION* (APPLICATION)	SOURCE	CATALOG NUMBER / CODE	SPECIFICITY
**CHOLINE ACETYL TRANSFERASE (CHAT)**	mouse	1:750 (DAB), 1:400-500 (fluorescent)	Dr. Costantino Cozzari		Characterized in [55]
**GABA**	rabbit	1:10 000 (postembedding immunogold)	Dr. Peter Somogyi	GABA9	Characterized in [56]
**GABA A RECEPTOR GAMMA 2 SUBUNIT**	rabbit	1:1000 (preembedding immunogold)	Synaptic Systems	224 003	Immunolabelling is abolished by virus-mediated gene-knock-out of GABAA receptors [57]
**GEPHYRIN**	mouse	1:100 (preembedding immunogold), 1:500 (fluorescent)	Synaptic Systems	147,021	Specific for the brain specific 93 kDa splice variant; KO verified (manufacturer's information)
**GREEN FLUORESCENT PROTEIN**	chicken	1:2000 (DAB, fluorescent)	Molecular Probes	A10262	no labelling in animals that were not injected with eGFP-expressing viruses
**NEUROLIGIN 2 (NL2)**	rabbit	1:600 (preembedding immunogold)	Synaptic Systems	129,203	No cross reactivity to neuroligins 1, 3, 4 (manufacturer's information); KO verified by our laboratory [1];
**TYPE 1 CANNABINOID RECEPTOR (CB1)**	goat	1:2000 (DAB)	Dr. Masahiko Watanabe		KO verified in [58]
**VESICULAR ACETYLCHOLINE TRANSPORTER (VACHT)**	rabbit	1:5000 (DAB, preembedding immunogold), 1:3000 (fluorescent)	Synaptic Systems	139 103	KO verified (manufacturer's information)
**VESICULAR ACETYLCHOLINE TRANSPORTER (VACHT)**	goat	1:10 000 (DAB, fluorescent)	Immunostar	1308002	Immunolabelling is completely abolished by preadsorption with synthetic rat VAT (vAChT) (511–530). Immunolabelling of transfected cells demonstrates no cross reactivity with vesicular monoamine transporters (manufacturer's information)

**VESICULAR GABA TRANSPORTER (VGAT)**	guinea-pig	1:2000 (fluorescent)	Synaptic Systems	131004	KO verified (manufacturer's information)
**GFP**	rabbit	1:2000 (fluorescent)	Molecular Probes	A11122	no labelling in animals that were not injected with eGFP-expressing viruses
**PV**	mouse	1:1000 (fluorescent)	Dr. Kenneth Baimbridge	-	stains the same as the KO-verified mouse ab
**PV**	rabbit	1:2000 (fluorescent)	SWANT	235	KO verified (manufacturer's information)
**GAD65**	mouse	1:250 (fluorescent)	Millipore (clone: GAD6)	MAB351	Recognizes the lower molecular weight isoform of the two GAD isoforms identified in brain [59].
**GEPHYRIN**	rabbit	1:2000 (fluorescent)	Synaptic Systems	147008 RbmAB7a	KO verified (manufacturer's information)
**VACHT**	guinea-pig	1:1000 (fluorescent)	Synaptic Systems	139 105	Gives identical staining to the K.O. verified 139 103 rabbit-anti-vAChT antibody
**SYNAPTO-PHYSIN**	mouse	1:1000 (fluorescent)	Sigma Aldrich, clone: SVP-38	S5768	[60]
**VESICULAR GLUTAMATE TRANSPORTER 1**	rabbit	1:1000 (fluorescent)	Synaptic Systems	135 302	KO verified (manufacturer's information)
* WHOLE SERUM WAS DILUTED OR STOCK SOLUTION WAS RECONSTITUTED AS DESCRIBED BY THE MANUFACTURER					

Supplementary Table 2 (secondary antibodies)

CONJUGATED WITH	RAISED IN	RAISED AGAINST	DILUTION* (PROCEDURE)	SOURCE	CATALOG NUMBER
**1.4-NM NANOGOLD**	goat	rabbit	1:100-1:300 (preembedding immunogold)	Nanoprobes	#2004
**10-NM GOLD**	goat	rabbit	1:1000 (postembedding immunogold)	BBI solutions	EM.GAR10
**ULTRA SMALL GOLD**	goat	mouse	1:50 (preembedding immunogold)	Aurion	100.022
**BIOTIN-SP**	donkey	goat	1:1000 (DAB)	JIL Inc. (Jackson Immunoresearch Laboratories, Inc.)	705-066-147
**BIOTIN-SP**	donkey	mouse	1:1000 (DAB)	JIL Inc.	715-066-151
**BIOTIN-SP**	donkey	goat	1:1000 (DAB)	JIL Inc.	705-066-147
**BIOTIN-SP**	donkey	rabbit	1:500 (DAB)	JIL Inc.	711-065-152
**BIOTIN**	goat	chicken	1:200 (DAB)	Vector Laboratories	BA-9010
**DYLIGHT 405**	donkey	mouse	1:200 Flow cytometry	JIL Inc.	715-475-151
**DYLIGHT 405**	donkey	rabbit	1:1000 (confocal)	JIL Inc.	711-475-152
**ALEXA 488**	goat	chicken	1:1000 (confocal), 1:500 (STORM)	Vector Laboratories	A-11039
**ALEXA 488**	donkey	rabbit	1:500 (confocal)	Invitrogen	A21206
**ALEXA 488**	donkey	chicken	1:1000 (confocal)	JIL Inc.	703-545-155
**ALEXA 488**	donkey	goat	1:1000 (confocal)	JIL Inc.	705-475-147
**CY-3**	donkey	mouse	1:500 (confocal)	JIL Inc.	715-165-151
**ALEXA 594**	donkey	rabbit	1:500 (confocal)	Invitrogen	A21207
**DYLIGHT 549**	donkey	guinea-pig	1:500 (confocal)	JIL Inc.	706-505-148
**ALEXA 594**	donkey	mouse	1:500 (confocal)	Life Technologies	A21203
**CF 568**	donkey	guinea-pig	1:500 (STORM)	Biotium	20377-500uL
**ALEXA 647**	donkey	guinea-pig	1:500 (confocal)	JIL Inc.	706-605-148
**CY-5**	donkey	goat	1:500 (confocal)	JIL Inc.	705-175-147
**ALEXA 647**	donkey	rabbit	1:500 (confocal, STORM)	JIL Inc.	711-605-152
**ALEXA 647**	donkey	mouse	1:500 (confocal)	JIL Inc.	715-605-151

Supplementary Table 3.,
The ratio of synapses of cholinergic fibres expressing gephyrin and $GABA_A$R γ2 subunit in different brain areas.

Mouse, area, labeling*	No. of synapses	No. of positive synapses	% of positive synapses
CA1, Gephyrin labelling in synapses of cholinergic fibres			
**WT3 ori (vAChT labelling)**	29	23	79
**WT3 pyr (vAChT labelling)**	5	5	100
**WT3 l-m (vAChT labelling)**	18	15	83
**WT4 ori (vAChT labelling)**	22	20	91
**WT4 pyr (vAChT labelling)**	2	2	100
**WT4 l-m (vAChT labelling)**	25	21	84
**ChAT-Cre1 ori (AAV-eYFP labelling)**	26	21	81
**ChAT-Cre1 pyr (AAV-eYFP labelling)**	1	1	100
**ChAT-Cre1 rad (AAV-eYFP labelling)**	22	15	68
**ChAT-Cre2 ori (AAV-eYFP labelling)**	37	29	78
**ChAT-Cre2 pyr (AAV-eYFP labelling)**	1	1	100
all	**188**	**153**	**81**
CA1, $GABA_A$R γ2 labelling in synapses of cholinergic fibres			
**WT3 l-m (vAChT labelling)**	31	24	77
**WT5 ori (vAChT labelling)**	27	20	74
**WT6 ori (vAChT labelling)**	16	11	69
**WT6 pyr (vAChT labelling)**	3	2	67
**WT6 l-m (vAChT labelling)**	20	16	80
**ChAT-Cre2 ori (AAV-eYFP labelling)**	32	27	84
**ChAT-Cre2 pyr (AAV-eYFP labelling)**	9	8	89
**ChAT-Cre2 rad (AAV-eYFP labelling)**	5	4	80
**ChAT-Cre3 ori (AAV-eYFP labelling)**	26	25	96
**ChAT-Cre3 pyr (AAV-eYFP labelling)**	3	1	33
all	**172**	**138**	**80**
S1, $GABA_A$Rγ2 labelling in synapses of cholinergic fibres			
**WT6 all (vAChT labelling)**	**36**	**30**	**83**

* WT3-6: indicates 4 wild type mice, ChAT-Cre1-3: indicates 3 different mice, ori: stratum oriens, pyr: stratum pyramidale, rad: stratum radiatum, l-m: stratum lacunosum-moleculare

Supplementary Table 4.
Measured parameters of reconstructed axonal segments

Axon ID a	Labelling (DAB-gold)	mouse	Length (µm)	No. of mitochondria	No. of synapses	Synapse density (syn./ 100 µm)	Postsynaptic targets: Pyramidal dendrite	Interneuron dendrite	Spine	Unidentified
A (in CA1 ori)	ChAT-NL2	WT1	22.0	4	12	54.5	8	1	3	0
C (in CA1 ori)	ChAT-NL2	WT2	21.5	3	8	37.2	3	1	4	0
D (in CA1 ori)	ChAT-NL2	WT2	14.8	4	5	33.8	5	0	0	0
E (in CA1 ori)	ChAT-NL2	WT2	6.8	1	4	58.5	2	1	0	1
F (in CA1 ori)	ChAT-NL2	WT2	10.0	4	6	60.1	5	1	0	0
B (in CA1 ori)	eYFP-gephyrin	ChAT-Cre1	38.1	11	15	39.4	10	0	3	2
M (in CA1 ori)	eYFP-gephyrin	ChAT-Cre1	27.0	7	10	37.0	6	1	0	3
N (in CA1 ori)	eYFP-gephyrin	ChAT-Cre2	12.3	2	4	32.6	4	0	0	0
O (in CA1 ori)	eYFP-gephyrin	ChAT-Cre2	18.4	4	6	32.7	4	0	2	0
**CA1 ori (cholinergic) all**			**170.9**	**40**	**70**	**41.0**	**47 (67.1%)**	**5 (7.1%)**	**12 (17.1%)**	**6 (8.6%)**
G (in CA1 rad)	ChAT-NL2	WT1	33.1	12	15	45.3	8	0	7	0
H (in CA1 rad)	ChAT-NL2	WT2	17.2	4	7	40.8	4	0	3	0
I (in CA1 rad)	ChAT-NL2	WT1	25.4	9	15	59.0	8	0	7	0
**CA1 rad (cholinergic) all**			**75.8**	**25**	**37**	**48.8**	**20 (54.1%)**	**0 (0%)**	**17 (45.9%)**	**0 (0%)**
**CA1 ori+rad (cholinergic) all**			**246.7**	**65**	**107**	**43.4**	**67 (62.6%)**	**5 (4.7%)**	**29 (27.1%)**	**6 (5.6%)**
P (in CA1 l-m)	ChAT-NL2	WT2	26.2	7	8	30.5	2[b]		5	1
Q (in CA1 l-m)	ChAT-NL2	WT2	17.0	5	6	35.3	2[b]		3	1
**CA1 l-m (cholinergic) all**			**43.2**	**12**	**14**	**32.4**	**4[b] (28.6%)**		**8 (57.1%)**	**2 (14.3%)**
**CA1 cholinergic fibers in all layers**			**289.9**	**77**	**121**	**41.7**	**76[b] (62.8%)**		**37 (30.6%)**	**8 (6.6%)**
J (in S1 LI)	ChAT-NL2	WT2	22.0	7	6	27.3	4[b]		2	0
K (in S1 LII/LIII)	ChAT-NL2	WT2	6.1	3	5	82.7	1[b]		3	1
L (in S1 LII)	ChAT-NL2	WT2	32.6	10	13	39.9	8[b]		4	1
**S1 cholinergic fibers in all layers**			**60.6**	**20**	**24**	**39.6**	**13[b] (54.2%)**		**9 (37.5%)**	**2 (8.3%)**
R (in CA1 ori)	CB_1-NL2	WT1	18.0	6	14	77.6	13	0	1	0
S (in CA1 rad)	CB_1-NL2	WT1	29.2	7	10	34.2	8	0	0	2
**CA1 CB_1-positive fibers in all layers**			**47.2**	**13**	**24**	**50.8**	**21 (87.5%)**	**0 (0%)**	**1 (4.2%)**	**2 (8.3%)**

[a]: WT3-6: indicates 4 wild type mice, ChAT-Cre1-3: indicates 3 different mice, ori: stratum oriens, pyr: stratum pyramidale, rad: stratum radiatum, l-m: stratum lacunosum-moleculare; [b]: We did not identify the cell types that established the dendritic shafts in CA1 lacunosum-moleculare and S1 (see Supplemental Experimental Procedures).

Supplementary References:

1. Takács, V. T., Freund, T. F. & Nyiri, G. Neuroligin 2 Is Expressed in Synapses Established by Cholinergic Cells in the Mouse Brain. *PLoS One* **8,** (2013).
2. Tyagarajan, S. K. & Fritschy, J.-M. Gephyrin: a master regulator of neuronal function? *Nat. Rev. Neurosci.* **15,** 141–156 (2014).
3. Dong, N., Qi, J. & Chen, G. Molecular reconstitution of functional GABAergic synapses with expression of neuroligin-2 and GABAA receptors. *Mol. Cell. Neurosci.* **35,** 14–23 (2007).
4. Poulopoulos, A. *et al.* Neuroligin 2 Drives Postsynaptic Assembly at Perisomatic Inhibitory Synapses through Gephyrin and Collybistin. *Neuron* **63,** 628–642 (2009).
5. Jedlicka, P. *et al.* Increased dentate gyrus excitability in neuroligin-2-deficient mice in vivo. *Cereb. Cortex* **21,** 357–367 (2011).
6. Essrich, C., Lorez, M., Benson, J. A., Fritschy, J.-M. & Lüscher, B. Postsynaptic clustering of major GABAA receptor subtypes requires the γ2 subunit and gephyrin. *Nat. Neurosci.* **1,** 563–571 (1998).
7. Schweizer, C. *et al.* The ??2 subunit of GABAA receptors is required for maintenance of receptors at mature synapses. *Mol. Cell. Neurosci.* **24,** 442–450 (2003).
8. Zhang, B., Ganetzky, B., Bellen, H. J. & Murthy, V. N. Tailoring uniform coats for synaptic vesicles during endocytosis. *Neuron* **23,** 419–422 (1999).
9. Karunanithi, S., Marin, L., Wong, K. & Atwood, H. L. Quantal Size and Variation Determined by Vesicle Size in Normal and Mutant Drosophila Glutamatergic Synapses. *J. Neurosci.* **22,** 10267–10276 (2002).
10. Takamori, S., Riedel, D. & Jahn, R. Immunoisolation of GABA-specific synaptic vesicles defines a functionally distinct subset of synaptic vesicles. *J. Neurosci.* **20,** 4904–4911 (2000).
11. Mutch, S. A. *et al.* Protein Quantification at the Single Vesicle Level Reveals That a Subset of Synaptic Vesicle Proteins Are Trafficked with High Precision. *J. Neurosci.* **31,** 1461–1470 (2011).
12. Qu, L., Akbergenova, Y., Hu, Y. & Schikorski, T. Synapse-to-synapse variation in mean synaptic vesicle size and its relationship with synaptic morphology and function. *J. Comp. Neurol.* **514,** 343–352 (2009).
13. Blusztajn, J. K. & Rinnofner, J. Intrinsic cholinergic neurons in the hippocampus: Fact or artifact? *Front. Synaptic Neurosci.* **8,** 6–11 (2016).
14. Descarries, L., Gisiger, V. & Steriade, M. Diffuse transmission by acetylcholine in the CNS. *Prog. Neurobiol.* **53,** 603–625 (1997).
15. Lendvai, B. & Vizi, E. S. Nonsynaptic Chemical Transmission Through Nicotinic Acetylcholine Receptors. *Physiol. Rev.* **88,** 333–349 (2008).
16. Vizi, E. S., Kiss, J. P. & Lendvai, B. Nonsynaptic communication in the central nervous system. *Neurochem. Int.* **45,** 443–451 (2004).
17. Zoli, M., Jansson, A., Syková, E., Agnati, L. F. & Fuxe, K. Volume transmission in the CNS and its relevance for neuropsychopharmacology. *Trends Pharmacol. Sci.* **20,** 142–150 (1999).
18. Vizi, E. S. Role of high-affinity receptors and membrane transporters in nonsynaptic communication and drug action in the central nervous system. *Pharmacol. Rev.* **52,** 63–89 (2000).
19. Agnati, L. F. *et al.* Volume transmission and wiring transmission from cellular to molecular networks: History and perspectives. *Acta Physiol.* **187,** 329–344 (2006).
20. Sarter, M., Parikh, V. & Howe, W. M. Phasic acetylcholine release and the volume transmission hypothesis: time to move on. *Nat. Rev. Neurosci.* **10,** 383–390 (2009).
21. De Lima, a D. & Singer, W. Cholinergic innervation of the cat striate cortex: a choline acetyltransferase immunocytochemical analysis. *J. Comp. Neurol.* **250,** 324–38 (1986).
22. Aznavour, N., Mechawar, N. & Descarries, L. Comparative analysis of cholinergic innervation in the dorsal hippocampus of adult mouse and rat: A quantitative immunocytochemical study. *Hippocampus* **12,** 206–217 (2002).

23. Umbriaco, D., Watkins, K. C., Descarries, L., Cozzari, C. & Hartman, B. K. Ultrastructural and Morphometric Features of the Acetylcholine Innervation in Adult-Rat Parietal Cortex - An Electron-Microscopic Study in Serial Sections. *J. Comp. Neurol.* **348,** 351–373 (1994).
24. Umbriaco, D., Garcia, S., Beaulieu, C. & Descarries, L. Relational features of acetylcholine, noradrenaline, serotonin and GABA axon terminals in the Stratum radiatum of adult rat hippocampus (CA1). *Hippocampus* **5,** 605–620 (1995).
25. Aznavour, N., Watkins, K. C. & Descarries, L. Postnatal development of the cholinergic innervation in the dorsal hippocampus of rat: Quantitative light and electron microscopic immunocytochemical study. *J. Comp. Neurol.* **486,** 61–75 (2005).
26. Yamasaki, M., Matsui, M. & Watanabe, M. Preferential Localization of Muscarinic M1 Receptor on Dendritic Shaft and Spine of Cortical Pyramidal Cells and Its Anatomical Evidence for Volume Transmission. *J. Neurosci.* **30,** 4408–4418 (2010).
27. Mrzljak, L., Pappy, M., Leranth, C. & Goldman???Rakic, P. S. Cholinergic synaptic circuitry in the macaque prefrontal cortex. *J. Comp. Neurol.* **357,** 603–617 (1995).
28. Smiley, J. F., Morrell, F. & Mesulam, M. M. Cholinergic synapses in human cerebral cortex: an ultrastructural study in serial sections. *Exp. Neurol.* **144,** 361–8 (1997).
29. Turrini, P. *et al.* Cholinergic nerve terminals establish classical synapses in the rat cerebral cortex: Synaptic pattern and age-related atrophy. *Neuroscience* **105,** 277–285 (2001).
30. Rouse, S. T., Edmunds, S. M., Yi, H., Gilmor, M. L. & Levey, A. I. Localization of M(2) muscarinic acetylcholine receptor protein in cholinergic and non-cholinergic terminals in rat hippocampus. *Neurosci Lett* **284,** 182–186 (2000).
31. Pepeu, G. & Giovannini, M. G. Changes in Acetylcholine Extracellular Levels During Cognitive Processes. 21–27 (2004). doi:10.1101/lm.68104.)
32. Parikh, V., Kozak, R., Martinez, V. & Sarter, M. Prefrontal Acetylcholine Release Controls Cue Detection on Multiple Timescales. *Neuron* **56,** 141–154 (2007).
33. Howe, W. M. *et al.* Prefrontal Cholinergic Mechanisms Instigating Shifts from Monitoring for Cues to Cue-Guided Performance: Converging Electrochemical and fMRI Evidence from Rats and Humans. *J. Neurosci.* **33,** 8742–8752 (2013).
34. Muñoz, W. & Rudy, B. Spatiotemporal specificity in cholinergic control of neocortical function. *Curr. Opin. Neurobiol.* **26,** 149–160 (2014).
35. Teles-Grilo Ruivo, L. M. *et al.* Coordinated Acetylcholine Release in Prefrontal Cortex and Hippocampus Is Associated with Arousal and Reward on Distinct Timescales. *Cell Rep.* **18,** 905–917 (2017).
36. Hangya, B., Ranade, S. P., Lorenc, M. & Kepecs, A. Central Cholinergic Neurons Are Rapidly Recruited by Reinforcement Feedback. *Cell* **162,** 1155–1168 (2015).
37. Letzkus, J. J. *et al.* A disinhibitory microcircuit for associative fear learning in the auditory cortex. *Nature* **480,** 331–335 (2011).
38. Gritton, H. J. *et al.* Cortical cholinergic signaling controls the detection of cues. *Proc. Natl. Acad. Sci.* **113,** E1089–E1097 (2016).
39. Gu, Z. & Yakel, J. L. Timing-Dependent Septal Cholinergic Induction of Dynamic Hippocampal Synaptic Plasticity. *Neuron* **71,** 155–165 (2011).
40. Allaway, K. C. & Machold, R. Developmental specification of forebrain cholinergic neurons. *Dev. Biol.* **421,** 1–7 (2017).
41. Fragkouli, A., van Wijk, N. V., Lopes, R., Kessaris, N. & Pachnis, V. LIM homeodomain transcription factor-dependent specification of bipotential MGE progenitors into cholinergic and GABAergic striatal interneurons. *Development* **136,** 3841–3851 (2009).
42. Bachy, I. & Rétaux, S. GABAergic specification in the basal forebrain is controlled by the LIM-hd factor Lhx7. *Dev. Biol.* **291,** 218–226 (2006).
43. Liodis, P. *et al.* Lhx6 Activity Is Required for the Normal Migration and Specification of Cortical Interneuron Subtypes. *J. Neurosci.* **27,** 3078–3089 (2007).

44. Fisher, R. S. & Levine, M. S. Transmitter cosynthesis by corticopetal basal forebrain neurons. *Brain Res.* **491,** 163–168 (1989).
45. Granger, A. J., Mulder, N., Saunders, A. & Sabatini, B. L. Cotransmission of acetylcholine and GABA. *Neuropharmacology* **100,** 40–46 (2016).
46. Beaulieu, C. & Somogyi, P. Enrichment of cholinergic synaptic terminals on GABAergic neurons and coexistence of immunoreactive GABA and choline acetyltransferase in the same synaptic terminals in the striate cortex of the cat. *J. Comp. Neurol.* **304,** 666–680 (1991).
47. Saunders, A., Granger, A. J. & Sabatini, B. L. Corelease of acetylcholine and GABA from cholinergic forebrain neurons. *Elife* **2015,** 1–13 (2015).
48. Pang, K. C. H., Jiao, X., Sinha, S., Beck, K. D. & Servatius, R. J. Damage of GABAergic neurons in the medial septum impairs spatial working memory and extinction of active avoidance: Effects on proactive interference. *Hippocampus* **21,** 835–846 (2011).
49. Henny, P. & Jones, B. E. Projections from basal forebrain to prefrontal cortex comprise cholinergic, GABAergic and glutamatergic inputs to pyramidal cells or interneurons. *Eur. J. Neurosci.* **27,** 654–670 (2008).
50. Münster-Wandowski, A., Gómez-Lira, G. & Gutiérrez, R. Mixed neurotransmission in the hippocampal mossy fibers. *Front. Cell. Neurosci.* **7,** 1–19 (2013).
51. Gundersen, V., Holten, A. T. & Storm-Mathisen, J. GABAergic synapses in hippocampus exocytose aspartate on to NMDA receptors: Quantitative immunogold evidence for co-transmission. *Mol. Cell. Neurosci.* **26,** 156–165 (2004).
52. Kljakic, O., Janickova, H., Prado, V. F. & Prado, M. A. M. Cholinergic/glutamatergic co-transmission in striatal cholinergic interneurons: New mechanisms regulating striatal computation. *J. Neurochem.* 1–13 (2017). doi:10.1111/jnc.14003
53. Lee, S., Kim, K. & Zhou, Z. J. Role of ACh-GABA Cotransmission in Detecting Image Motion and Motion Direction. *Neuron* **68,** 1159–1172 (2010).
54. Saunders, A. *et al.* A direct GABAergic output from the basal ganglia to frontal cortex. *Nature* **521,** 85–89 (2015).
55. Cozzari, C., Howard, J. & Hartman, B. Analysis of epitopes on choline acetyltransferase (ChAT) using monoclonal antibodies. in *Soc Neurosci Abstr 16* 200 (1990).
56. Somogyi, P. & Hodgson, A. J. Antisera to gamma-aminobutyric acid. III. Demonstration of GABA in Golgi-impregnated neurons and in conventional electron microscopic sections of cat striate cortex. *J. Histochem. Cytochem.* **33,** 249–257 (1985).
57. Rovo, Z. *et al.* Phasic, Nonsynaptic GABA-A Receptor-Mediated Inhibition Entrains Thalamocortical Oscillations. *J. Neurosci.* **34,** 7137–7147 (2014).
58. Uchigashima, M. *et al.* Subcellular Arrangement of Molecules for 2-Arachidonoyl-Glycerol-Mediated Retrograde Signaling and Its Physiological Contribution to Synaptic Modulation in the Striatum. *J. Neurosci.* **27,** 3663–3676 (2007).
59. Gottlieb, I. Characterization of the Proteins Purified with Monoclonal to Glutamic Acid Decarboxylase. **8,** 2123–2130 (1988).
60. Obata, K. *et al.* Four synaptic vesicle-specific proteins: identification by monoclonal antibodies and distribution in the nervous tissue and the adrenal medulla. *Brain Res.* **404,** 169–179 (1987).
